# Strain engineering and metabolic flux analysis of a probiotic yeast *Saccharomyces boulardii* for metabolizing l-fucose, a mammalian mucin component

**DOI:** 10.1186/s12934-022-01926-x

**Published:** 2022-10-07

**Authors:** Jungyeon Kim, Yu Eun Cheong, Sora Yu, Yong-Su Jin, Kyoung Heon Kim

**Affiliations:** 1 Carl R. Woese Institute for Genomic Biology, University of Illinois at Urbana-Champaign, Urbana, IL 61801 USA; 2 Department of Biotechnology, Graduate School, Korea University, Seoul, 02841 Republic of Korea; 3 Department of Food Science and Human Nutrition, University of Illinois at Urbana-Champaign, Urbana, IL 61801 USA; 4 Department of Food Bioscience and Technology, College of Life Sciences and Biotechnology, Korea University, Seoul, 02841 Republic of Korea

**Keywords:** l-Fucose, *Saccharomyces boulardii*, *Saccharomyces cerevisiae*, Genome-scale metabolic model analysis, Elementary flux mode analysis

## Abstract

**Background:**

*Saccharomyces boulardii* is a probiotic yeast that exhibits antimicrobial and anti-toxin activities. Although *S. boulardii* has been clinically used for decades to treat gastrointestinal disorders, several studies have reported weak or no beneficial effects of *S. boulardii* administration in some cases. These conflicting results of *S. boulardii* efficacity may be due to nutrient deficiencies in the intestine that make it difficult for *S. boulardii* to maintain its metabolic activity.

**Results:**

To enable *S. boulardii* to overcome any nutritional deficiencies in the intestine, we constructed a *S. boulardii* strain that could metabolize l-fucose, a major component of mucin in the gut epithelium. The *fucU*, *fucI*, *fucK*, and *fucA* from *Escherichia coli* and *HXT4* from *S. cerevisiae* were overexpressed in *S. boulardii*. The engineered *S. boulardii* metabolized l-fucose and produced 1,2-propanediol under aerobic and anaerobic conditions. It also produced large amounts of 1,2-propanediol under strict anaerobic conditions. An in silico genome-scale metabolic model analysis was performed to simulate the growth of *S. boulardii* on l-fucose, and elementary flux modes were calculated to identify critical metabolic reactions for assimilating l-fucose. As a result, we found that the engineered *S. boulardii* consumes l-fucose via *(S)*-lactaldehyde-*(S)*-lactate-pyruvate pathway, which is highly oxygen dependent.

**Conclusion:**

To the best of our knowledge, this is the first study in which *S. cerevisiae* and *S. boulardii* strains capable of metabolizing l-fucose have been constructed. This strategy could be used to enhance the metabolic activity of *S. boulardii* and other probiotic microorganisms in the gut.

**Supplementary Information:**

The online version contains supplementary material available at 10.1186/s12934-022-01926-x.

## Background

*Saccharomyces boulardii* is a probiotic yeast known to interact with its host and exhibits antimicrobial activity [1, 2], antitoxin effects [3, 4], and immunoregulatory effects in the human intestine [5]. *S. boulardii* has a genome similar to that of *S. cerevisiae* but has a distinct phenotype [6]. For example, *S. boulardii* can readily grow at 37 °C, the normal human body temperature, and attach to human [7] or mouse intestinal epithelial cells [7, 8]. In addition, *S. boulardii* is more resistant to environmental stressors such as heat and acid than *S. cerevisiae* [9]. Because of these growth advantages, *S. boulardii* has higher metabolic activity in the intestine than *S. cerevisiae* and provides various health benefits in humans [6]. In particular, *S. boulardii* has prophylactic and therapeutic effects for many gastrointestinal diseases caused by pathogens such as pathogenic *Escherichia coli* [10, 11], *Salmonella enterica* serotype Typhimurium [12], *Campylobacter jejuni* [13, 14], and *Clostridium difficile* [3, 15]. These therapeutic effects have been reported in numerous clinical trials, and *S. boulardii* has been used for decades as an over-the-counter medicine to treat or prevent diarrhea in Europe, Africa, and America [16–18].

Although the therapeutic effects of *S. boulardii* administration have been validated in many studies, several studies have reported that *S. boulardii* administration had weak or no beneficial effects [19]. For example, Canani et al. reported that the administration of *Lactobacillus rhamnosus* GG or a mixture of probiotic bacteria reduced disease duration in children suffering from acute diarrhea, but the administration of *S. boulardii* had no effect [20]. In addition, Lewis et al. reported that *S. boulardii* administration to the elderly suffering from diarrhea was not effective in reducing the levels of the *C. difficile* toxin or treating general diarrhea [21]. Furthermore, Surawicz et al. reported that the administration of low-dose vancomycin and *S. boulardii* was ineffective in the treatment of *C. difficile* infection, although administration of high-dose vancomycin and *S. boulardii* was more effective than vancomycin alone [22]. These conflicting reports on the therapeutic effects of *S. boulardii* administration may be due to its limited ability to grow or maintain its normal metabolic activity in the intestine. In general, *S. boulardii* can remain in human and mouse intestines only after oral administration at a high dose, and it is completely excreted 3–7 days post-administration [23–25]. Interestingly, *S. boulardii* has been reported to colonize intestinal epithelial cells in antibiotic-treated humans or gnotobiotic mice [7, 8, 22, 26]. This suggests that although *S. boulardii* can grow and exhibit various metabolic activities in the intestine, it can be easily inhibited by competition with other gut microorganisms [6]. The reduced viability or metabolic activity of *S. boulardii* in the intestinal environment may cause the probiotic yeast to be degraded or excreted from the intestine before its therapeutic effects can be realized.

After colonizing the host, gut microorganisms metabolize molecules that are either secreted by the host or part of its diet [27]; however, dietary nutrients are competitively consumed by the host and other gut microorganisms [28, 29]. To overcome insufficient nutrient supply, some microorganisms metabolize mucins secreted by mammalian hosts [27]. Mucin is mainly composed of l-fucose, d-galactose, *N*-acetylgalactosamine, *N*-acetylglucosamine, d-glucuronic acid, and sialic acid [30]. Of these, gut microorganisms most frequently use l-fucose as a carbon source [31, 32]. Microorganisms such as *Bacteroides thetaiotaomicron, Bacteroides fragilis*, and *Bifidobacterium bifidum* secrete fucosidase to cleave l-fucose from the glycoprotein terminus of mucin molecules [33, 34]. The free l-fucose is then metabolized by other microbes via the fucose kinase pathway [35]. In this pathway, fucose mutarotase (*fucU*) converts β-l-fucose to α-l-fucose at the initial phase. Then, fucose isomerase (*fucI*) converts α-l-fucose to l-fuculose, which is then phosphorylated to l-fuculose-1-phosphate by fuculose kinase (*fucK*); this is followed by the conversion of fuculose-1-phosphate to lactaldehyde (LAD) and dihydroxyacetophenone (DHAP) by the action of fuculose phosphate aldolase (*fucA*) [35]. DHAP is used to synthesize building blocks for cell growth, while LAD is converted to 1,2-propanediol (1,2-PDO), which is converted to propionate by other microorganisms such as *Eubacterium hallii* [29]. 1,2-PDO is safe to humans as it is registered as generally recognized as safe (GRAS), and propionate is a short-chain fatty acid that is beneficial to humans [29]. This survival strategy can be applied to *S. boulardii* to ensure that it has access to an energy source in the intestine.

Our goal was to construct an engineered *S. bouladii* strain that can metabolize l-fucose for improved survival and metabolic activities in the intestine. Although the same genetic engineering techniques may be used for *S. boulardii* and *S. cerevisiae*, their efficiency is at least 30- to 50-fold lower in *S. boulardii* [36]; hence, we first introduced a fucose metabolic pathway using *S. cerevisiae* as a testbed. To investigate whether fucose metabolism in yeast strains can support cell growth, we performed in silico simulations using a genome-scale metabolic model (GEM) of *S. cerevisiae* (yeast-GEM; version 8.5.0) [37]. In addition, we used elementary flux mode analysis (EFMA) [38] to infer more details about the metabolic fluxes that involve l-fucose and generate energy under aerobic and anaerobic conditions.

## Results and discussion

### Growth simulation of *S. cerevisiae* that can metabolize l-fucose based on GEM analysis

**Fig. 1:**
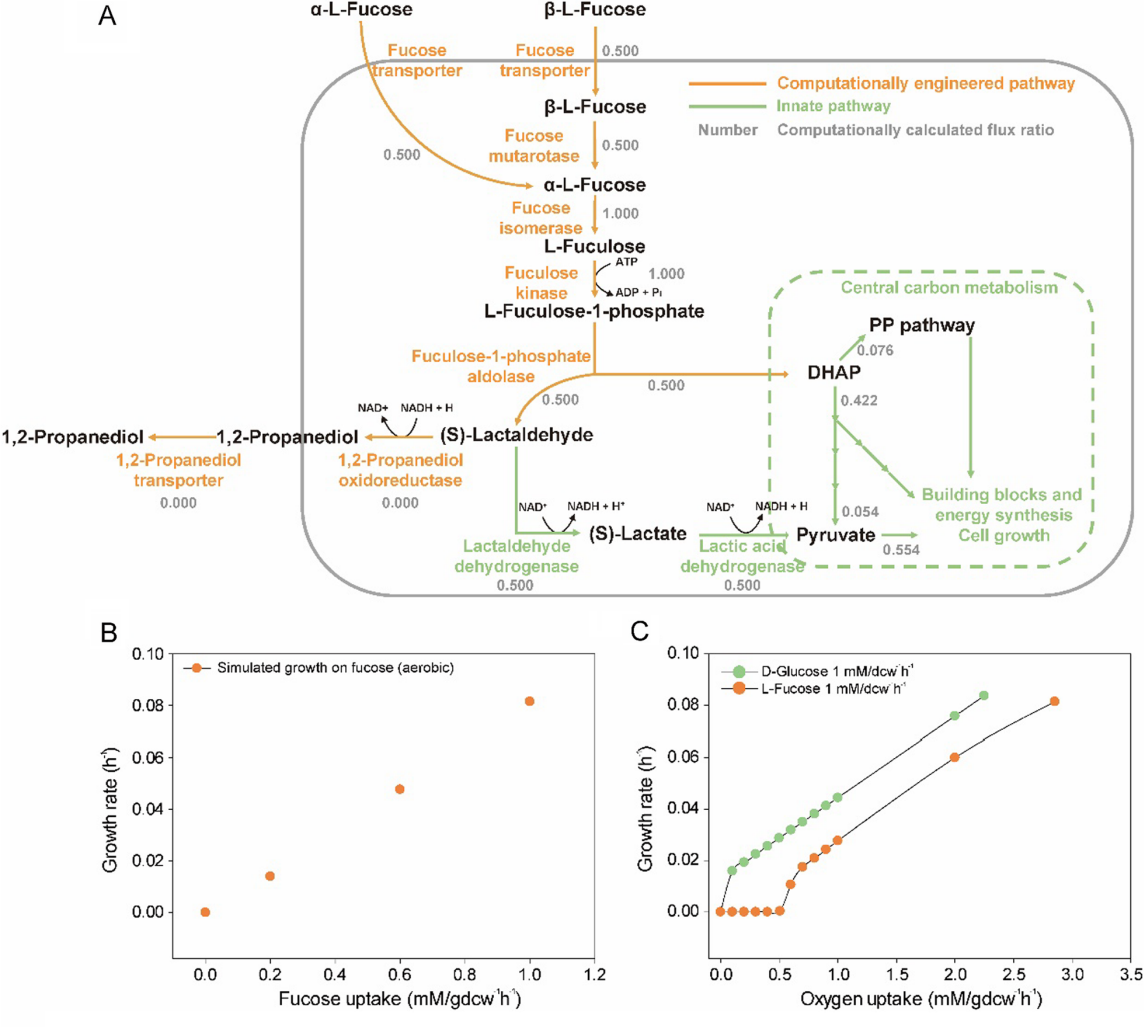
In silico genome-scale metabolic model analysis for the growth of *Saccharomyces cerevisiae* during l-fucose metabolism. **A** Fucose metabolism for cellular growth and detailed stoichiometry of each reaction. **B** In silico calculation of fucose uptake and growth rates, **C** Relationship between oxygen supply and growth on d-glucose or l-fucose

Microorganisms that metabolize l-fucose must synthesize biological building blocks from lower glycolysis intermediates such as DHAP; thus, they are characterized by low energy levels and a slow growth rate [39]. To confirm that yeast can synthesize sufficient amounts of biological building blocks for cell growth while metabolizing l-fucose, we simulated in silico growth using a *S. cerevisiae* GEM (Fig. 1 and Additional file 1). During the growth simulation under aerobic conditions (objective function: biomass synthesis; oxygen supply unlimited), *S. cerevisiae* metabolized l-fucose at a rate of 1 mmol/g dried cell weight (DCW)/h and showed a specific growth rate of 0.08145/h (Fig. 1B). DHAP, which is an important intermediate in fucose metabolism and is also a part of the pentose phosphate pathway and glycolysis, is used for the synthesis of biological building blocks (Fig. 1A). LAD, another key intermediate in fucose metabolism, is known to be converted to 1,2-PDO [39]; however, in the growth simulation, all of the LAD was converted to pyruvate by lactaldehyde dehydrogenase and lactic acid dehydrogenase and used for the production of biological building blocks (Fig. 1A). In addition, the simulated *S. cerevisiae* secreted ethanol while metabolizing d-glucose but did not secrete extracellular metabolites such as ethanol, glycerol, and acetate while metabolizing l-fucose.

Because the concentrations of oxygen vary in each part of the intestine, the amount of oxygen required to aerobically metabolize a carbon source is an important factor [40]. To infer how efficiently the yeast metabolizes l-fucose in the intestine, the oxygen requirements for the growth of l-fucose metabolizing yeast were compared to those for d-glucose metabolizing yeast (Fig. 1C). When 1 mmol of each carbon source was metabolized, yeast metabolizing d-glucose required less than 0.1 mmol oxygen for growth, whereas yeast metabolizing l-fucose required at least 0.507 mmol oxygen (Fig. 1C). These results show that the growth of yeast that can metabolize l-fucose is more oxygen-dependent than that of yeast metabolizing d-glucose. Under anaerobic conditions, *S. boulardii* consumes glucose and efficiently synthesizes energy and building blocks via pentose phosphate (PP) pathway and amino acid synthesis without gluconeogenesis. On the other hand, the two intermediates produced by fucose metabolism are DHAP and pyruvate, the initial and final molecules of lower glycolysis, so *S. boulardii* that metabolizes l-fucose need to building blocks via gluconeogenesis [39]. Under aerobic conditions, pyruvate is used for energy production through TCA cycle and DHAP is used for the synthesis of building blocks, so carbon fluxes of fucose metabolism can be more efficient than. Under anaerobic conditions where TCA cycle is unavailable and more DHAP is introduced into the glycolytic flux to synthesize energy resulting in reduced metabolic flux to synthesize building blocks. In addition, activation of the *(S)*-lactaldehyde-*(S)*-lactate-pyruvate pathway is difficult due to the lack of an oxidizing agent (Fig. 1A). In summary, fucose metabolism is highly oxygen dependent due to inefficient carbon fluxes and cofactor imbalance.

### Construction of a *S. cerevisiae* strain that can metabolize l-fucose

**Fig. 2:**
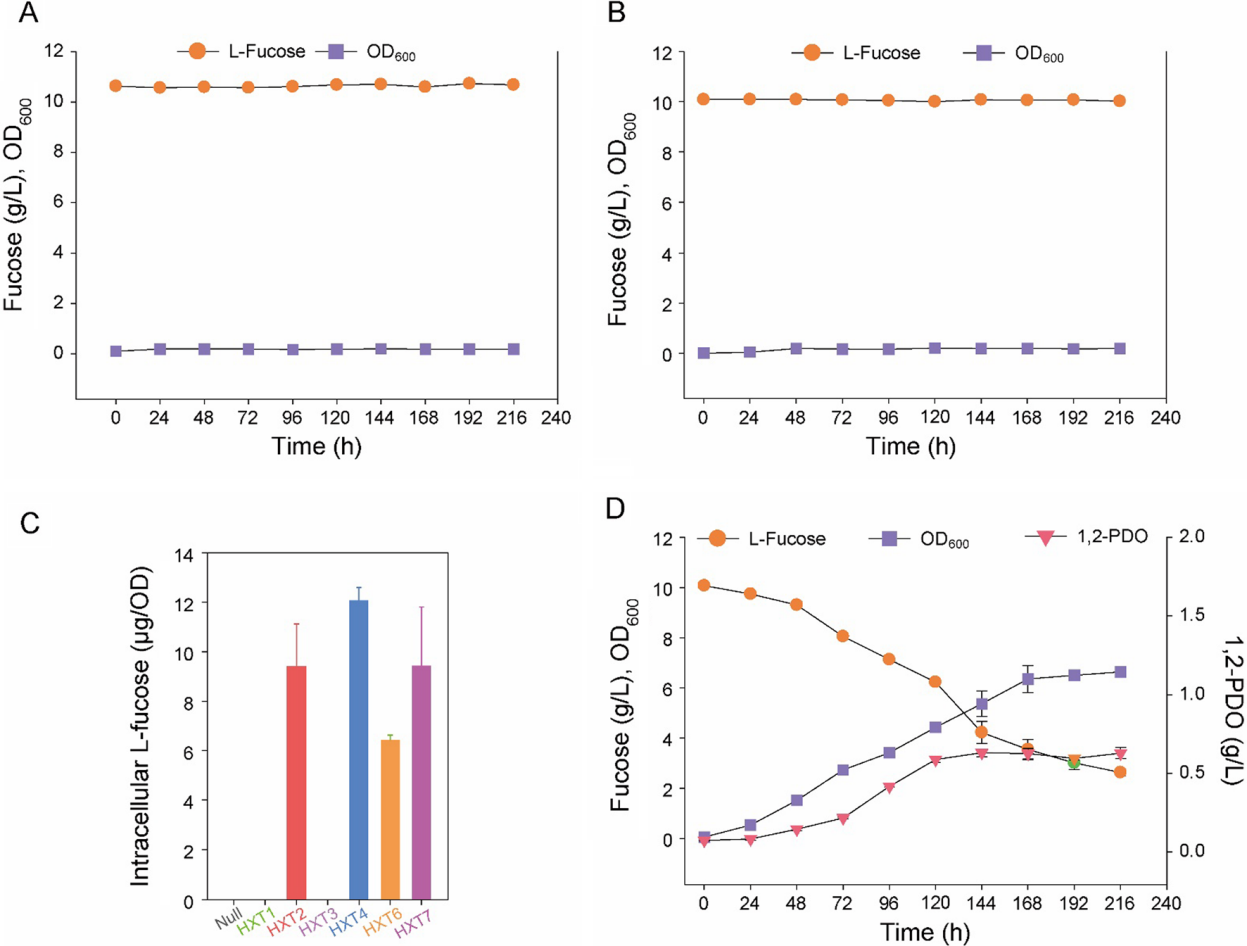
Growth profiles of *Saccharomyces cerevisiae* while metabolizing l-fucose and comparisons of fucose transport efficiency. **A** Growth profile of wild-type *S. cerevisiae*, **B** Growth profile of *S. cerevisiae* FC, **C** Intracellular l-fucose concentrations in the HXT-Null strain and HXT1-7 overexpressed strains. **D** Growth profile of *S. cerevisiae* FCT. Three biological replicates were used

The GEM model showed that the expression of undesignated fucose transporter, fucose mutarotase (*fucU*), fucose isomerase (*fucI*), fuculose kinase (*fucK*), and fuculose-1-phosphate aldolase (*fucA*) in *S. cerevisiae* was sufficient to allow the cells to metabolize l-fucose for growth (Fig. 1). In a previous study, Yu et al. reported that *S. cerevisiae* can transport small amounts of l-fucose [41]. In addition, the fucose permease (*fucP*) from the prokaryotic *E. coli* is less likely to be expressed in the eukaryotic *S. cerevisiae*. Therefore, except the fucose transporter, *fucU*, *fucI*, *fucK*, and *fucA* from *E. coli* K12 MG1655 were overexpressed in *S. cerevisiae* CEN. PK strain (*S. cerevisiae* FC; Table 1). However, neither wild-type *S. cerevisiae* (*S. cerevisiae* WT) nor *S. cerevisiae* FC was able to metabolize l-fucose in the yeast nitrogen base (YNB) medium (Fig. 2A, B). This could be because the yeast cells could not import enough l-fucose. To identify an appropriate transporter, we compared the l-fucose transport efficiency of an HXT-null *S. cerevisiae* expressing each hexose transporter (Table 1) [42]. We found that *HXT2*, *HXT4*, *HXT6*, or *HXT7* expressing strains were able to transport l-fucose, and that the *HXT4* strain had the highest fucose transport ability (Fig. 2C); therefore, we constructed a *S. cerevisiae* FCT strain that overexpressed fucose catabolic genes and *HXT4* (Table 1). Under aerobic conditions, *S. cerevisiae* FCT metabolized l-fucose (0.27 ± 0.018 mmol fucose/g DCW/h) in the YNB medium. This strain was also able to grow on l-fucose (specific growth rates: 0.034 ± 0.001/h) and produced 1,2-PDO (0.045 ± 0.004 mmol 1,2-PDO/g DCW/h) (Fig. 2D). According to the GEM results, *S. cerevisiae* showed optimal growth when LAD was converted to lactate rather than 1,2-PDO (Fig. 1A); therefore, 1,2-PDO oxidoreductase was not overexpressed in *S. cerevisiae* FCT. The production of 1,2-PDO without the overexpression of 1,2-PDO oxidoreductase indicates that *S. cerevisiae* has an unidentified or promiscuous 1,2-PDO oxidoreductase that is expressed during fucose metabolism.

**Table 1:** Strains, plasmids, and primers used in this study

Strain/plasmid/primer	Description	References/source
Strains		
*E. coli* DH5α	F − ϕ80d, *lac*Z*ΔM15*, *endA1*, *recA1*, *hsdR17*(rK − mK−), *supE44*, *thi-1*, *gyrA96*, *relA1*, Δ(*lacZYA*-*argF*)U169	Invitrogen
*S. cerevisiae* CEN.PK2-1D	*MAT*α *ura3-52 leu2-3*,*112 trp1-289 his3*Δ *MAL2-8c SUC2*	
*S. cerevisiae* WT	*S. cerevisiae* CEN.PK2-1D harboring pRS423GPD, pRS424GPD, pRS426GPD	This study
*S. cerevisiae* FC	*S. cerevisiae* CEN.PK2-1D harboring pRS423GPD_*fucA*, pRS424GPD_*fucIU*, pRS426GPD*_fucK*	This study
*S. cerevisiae* FCT	*S. cerevisiae* CEN.PK2-1D harboring pRS423GPD_*fucA*, pRS424GPD_*fucIU*, pRS426GPD*_fucK_HXT4*	This study
*S. boulardii* (ATCC MYA-796)	*MAT*α *ura3-52 leu2-3*,*112 trp1-289 his3*Δ *MAL2-8c SUC2*	[36]
*S. boulardii* WT	*S. boulardii* harboring pRS423GPD, pRS424GPD, pRS426GPD	This study
*S. boulardii* FCT	*S. boulardii* harboring pRS423GPD_*fucA*, pRS424GPD_*fucIU*, pRS426GPD*_fucK_HXT4*	This study
HXT-null *S. cerevisiae*	*S. cerevisiae* D452-2 with a xylose pathway and deletion of *HXT1*-*HXT7*	[42]
*S. cerevisiae HXT1*	Hxt-null *S. cerevisiae* with overexpression of *HXT1*	[42]
*S. cerevisiae HXT2*	Hxt-null *S. cerevisiae* with overexpression of *HXT2*	[42]
*S. cerevisiae HXT3*	Hxt-null *S. cerevisiae* with overexpression of *HXT3*	[42]
*S. cerevisiae HXT4*	Hxt-null *S. cerevisiae* with overexpression of *HXT4*	[42]
*S. cerevisiae HXT6*	Hxt-null *S. cerevisiae* with overexpression of *HXT6*	[42]
*S. cerevisiae HXT7*	Hxt-null *S. cerevisiae* with overexpression of *HXT7*	[42]
Plasmids		
prs423GPD	*HIS3*, GPD promoter, CYC1 terminator, 2 µ origin, and Ampr	[43]
prs424GPD	*LEU2*, GPD promoter, CYC1 terminator, 2 µ origin, and Ampr	[43]
prs426GPD	*URA3*, GPD promoter, CYC1 terminator, 2 µ origin, and Ampr	[43]
prs423GPD_*fucA*	pRS423GPD harboring *fucA* from *E. coli* K12 MG1655	This study
prs424GPD_*fucIfucU*	pRS424GPD harboring *fucI* and *fucU* from *E. coli* K12 MG1655	This study
prs426GPD_*fucKHXT4*	pRS426GPD harboring *fucK* from *E. coli* K12 MG1655 and *HXT4* from *S. cerevisiae* CEN.PK2-1D	This study
prs426GPD_*fucK*	pRS426GPD harboring *fucK* from *E. coli* K12 MG1655	This study
Primers		
F_Eco_*fucA*	5′-ACTAGTATGGAACGAAATAAACTTGCTC-3′	This study
R_Eco_*fucA*	5′-CTCGAGTTACTCTTCAATTCGTAACC-3′	This study
F_Eco_*fucI*	5′-ACTAGTATGAAAAAAATCAGCTTACCGAA-3′	This study
R_Eco_*fucI*	5′-CTCGAGTTAACGCTTGTACAACGGAC-3′	This study
F_Eco_*fucU*	5′-ACTAGTATGCTGAAAACAATTTCGCC-3′	This study
R_Eco_*fucU*	5′-CTCGAGTTACGGTGTTACCCCTTTTT-3′	This study
F_Eco_*fucK*	5′-ACTAGTATGAAACAAGAAGTTATCCTGG-3′	This study
R_Eco_*fucK*	5′-CTCGAGTCACACTTCCTCTATAAATT-3′	This study
F_Sce_*HXT4*	5′-ACTAGTATGTCTGAAGAAGCTGCCTATCAA-3′	This study
R_Sce_*HXT4*	5′CTCGAGCTACTTTTTTCCGAACATCT-3′	This study

### Fermentation profiles of *S. boulardii* metabolizing l-fucose under different oxygen concentrations

To construct an engineered *S. boulardii* strain that can metabolize l-fucose, the genes necessary for fucose catabolism and *HXT4* were overexpressed in *S. boulardii* (*S. boulardii* FCT; Table 1). Under aerobic conditions, wild-type *S. boulardii* (*S. boulardii* WT) was not able to metabolize l-fucose in YNB medium, whereas *S. boulardii* FCT was able to metabolize l-fucose (0.209 ± 0.004 mmol fucose/g DCW/h) and produce biomass (growth rate: 0.047 ± 0.001/h) and 1,2-PDO (0.018 ± 0.003 mmol 1,2-PDO/g DCW/h). During the first 72 h, *S. boulardii* FCT metabolized 14.69 ± 0.98 mmol fucose and produced 1.19 ± 0.16 mmol 1,2-PDO. *S. boulardii* FCT consumed the same amount of fucose as *S. cerevisiae* FCT and produced less 1,2-PDO and more biomass. Although the genomes of *S. cerevisiae* and *S. boulardii* are almost identical, it is known that the metabolism is slightly different due to mutations of various genes. For example, due to a point mutation in *PGM2*, *S. boulardii* metabolizes galactose very slowly [36] unlike *S. cerevisiae* which can metabolizes galactose rapidly. The overall metabolic changes induced by unknown mutations in those yeast strains might have led to different biomass and 1,2-PDO synthesis between *S. cerevisiae* FCT and *S. boulardii* FCT.

To examine the fucose metabolism of *S. boulardii* FCT in the intestinal environment, where the supply of oxygen is limited, we investigated its growth profiles under microaerobic and anaerobic conditions. Under microaerobic conditions, *S. boulardii* metabolized l-fucose (0.081 ± 0.001 mmol fucose/g DCW/h) and produced biomass (growth rate: 0.002 ± 0.0002/h) and 1,2-PDO (0.050 ± 0.004 mmol/g DCW/h). During the first 216 h, *S. boulardii* FCT metabolized 7.70 ± 0.88 mmol fucose and produced 2.89 ± 0.20 mmol 1,2-PDO. Under strict anaerobic conditions, *S. boulardii* metabolized l-fucose (0.045 ± 0.014 mmol fucose/g DCW/h) and produced 1,2-PDO (0.033 ± 0.003 mmol/g DCW/h) without growth. During the first 72 h, *S. boulardii* FCT metabolized 3.43 ± 0.27 mmol fucose and produced 1.83 ± 0.07 mmol 1,2-PDO. Because the oxygen supply was limited, yeast synthesized 1,2-PDO instead of biomass.

**Fig. 3:**
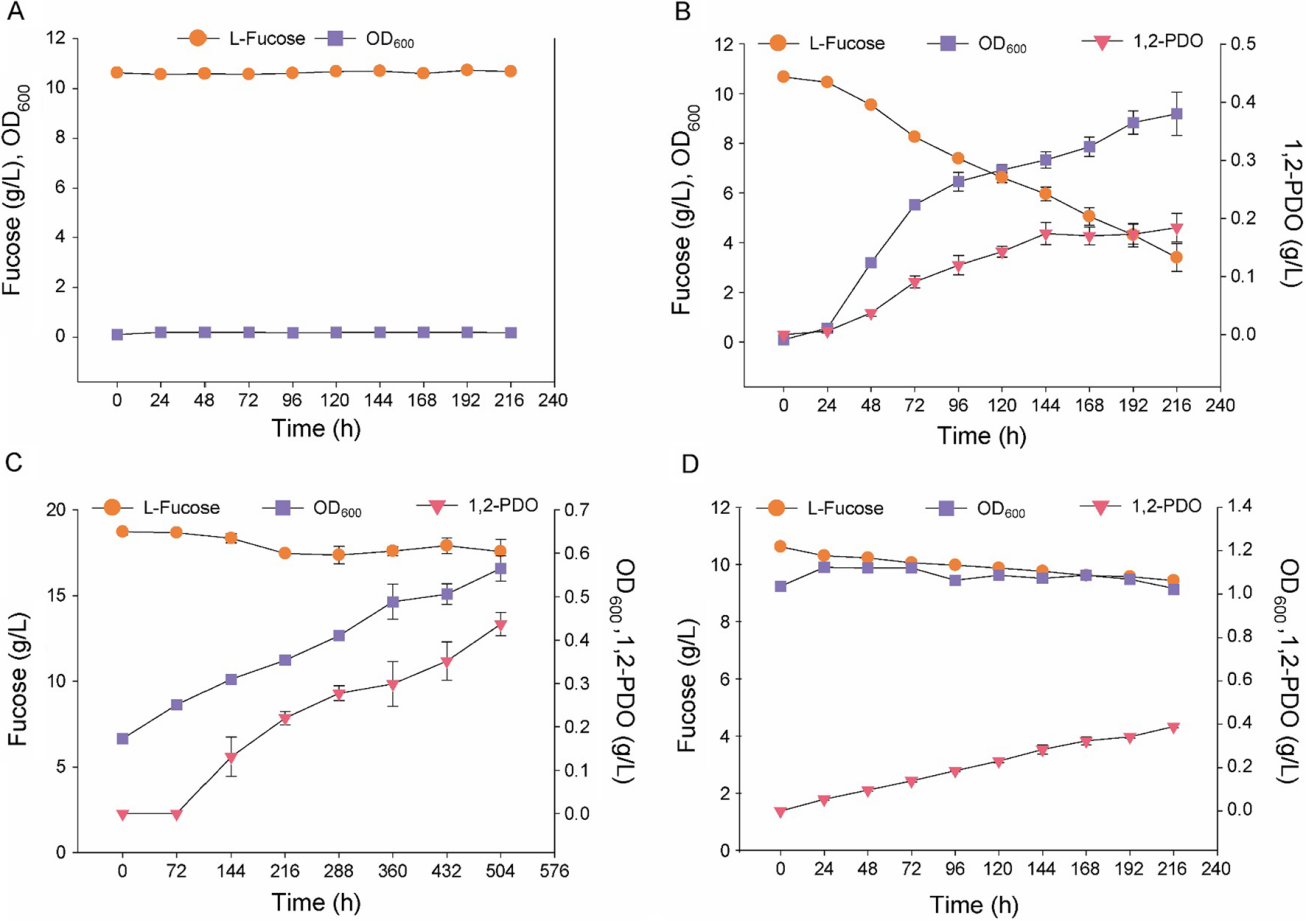
Growth profiles of *Saccharomyces boulardii* while metabolizing l-fucose under different aerobic conditions. **A** Growth profiles of wild-type *S. boulardii*, **B** Growth profiles of *S. boulardii* FCT under aerobic conditions, **C** Growth profiles of *S. boulardii* FCT under microaerobic conditions, **D** Growth profiles of *S. boulardii* FCT under strict anaerobic conditions. Three biological replicates were used

The high oxygen dependence of the *(S)*-lactaldehyde-*(S)*-lactate-pyruvate pathway inhibits the growth of *S. boulardii* FCT when it metabolizes l-fucose under anaerobic conditions. The GEM results revealed that the key steps for fucose-dependent yeast growth were the conversion of LAD to *(S)*-lactate and then to pyruvate using two oxidizing agents (Fig. 1A). In addition, we found that yeast growth based on fucose metabolism is highly oxygen-dependent because the *(S)*-lactaldehyde-*(S)*-lactate-pyruvate pathway requires many oxidizing agents (Fig. 1C). In the growth profile of *S. boulardii* FCT, which can metabolize l-fucose, the growth slowed markedly under microaerobic conditions and completely stopped under strict anaerobic conditions (Fig. 3C, D). Interestingly, *S. boulardii* FCT was able to metabolize l-fucose, even in an oxygen-limited environment, and showed a tendency to synthesize 1,2-PDO rather than biomass. These results suggest that under the conditions of limited oxygen supply, *S. boulardii* FCT metabolizes l-fucose through a specific metabolic pathway distinct from that predicted by the GEM.

### Prediction of yeast fucose metabolism under aerobic and anaerobic conditions using EFMA

**Fig. 4:**
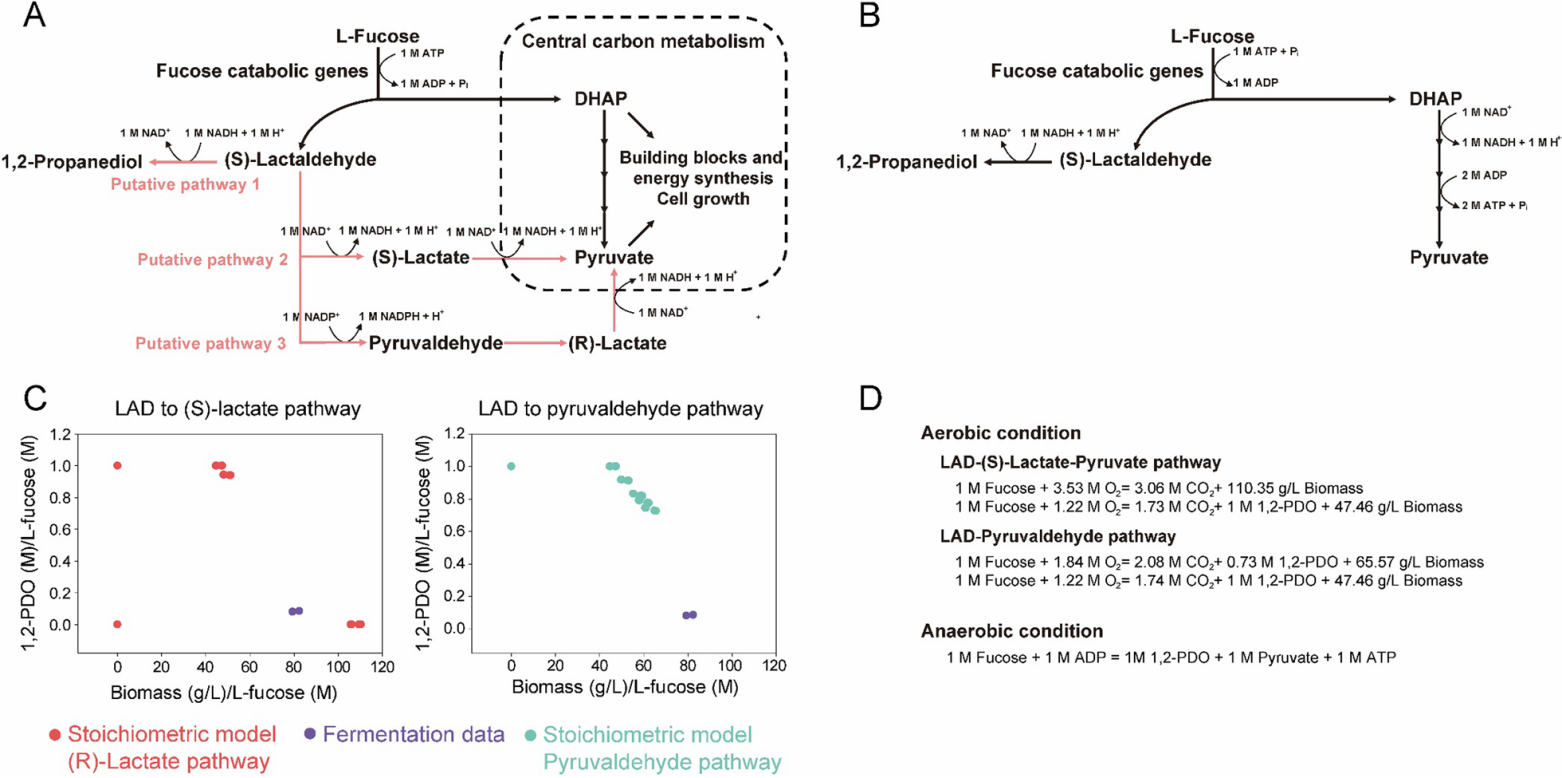
Elementary flux mode analysis for elucidating fucose metabolism under aerobic and anaerobic conditions. **A** Schematic diagram for putative fucose metabolism under aerobic conditions (**B**) and under anaerobic conditions. **C** Amount of 1,2-PDO and biomass produced by metabolizing fucose under aerobic conditions, **D** Stoichiometry for the maximum production of 1,2-PDO, biomass or energy in each condition

The GEM results predicted the growth profile of *S. boulardii* FCT under aerobic conditions to be similar to that of the fermentation profile but did not predict the synthesis of 1,2-PDO (Fig. 3B). In addition, when the supply of oxygen was limited, *S. boulardii* FCT still metabolized l-fucose but produced 1,2-PDO instead of biomass (Fig. 3C, D). These results suggest that the fucose metabolism pathways predicted by the GEM and the cellular fucose metabolism pathways are different, especially in an oxygen-limited environment. We used EFMA to investigate the fucose metabolic pathways that allow the yeast to grow and produce energy along with small amounts of 1,2-PDO under aerobic conditions and to produce energy and large amounts of 1,2-PDO under anaerobic conditions (Fig. 4). DHAP and LAD are key intermediates in fucose metabolism. DHAP is also an intermediate in glycolysis and can be directly used for the synthesis of energy and building blocks (Fig. 4A). On the other hand, LAD can be used for the synthesis of building blocks only after it is converted to pyruvate by the *(S)*-lactaldehyde-*(S)*-lactate-pyruvate pathway [LAD to *(S)*-lactate pathway] or the *(S)*-lactaldehyde-pyruvaldehyde®lactate-pyruvate pathway (LAD to pyruvaldehyde pathway; Fig. 1A). The pathways that convert LAD to pyruvate require oxidative power (Fig. 1A). Alternatively, reductive power can be used to directly convert LAD to 1,2-PDO (Fig. 1A).

A set of elementary flux modes to produce biomass and 1,2-PDO using each pathway under aerobic conditions was investigated (Fig. 4A). The LAD to *(S)*-lactate pathway had 39 models, and the LAD to pyruvaldehyde pathway had 51 models (Fig. 4C). In the LAD to *(S)*-lactate pathway, 1 M l-fucose was metabolized to synthesize up to 110.35 g/L biomass or up to 1 M 1,2-PDO and 47.46 g/L biomass (Fig. 4C, D). In the LAD to pyruvaldehyde pathway, 1 M l-fucose was metabolized to produce up to 0.73 M 1,2-PDO and 65.57 g/L biomass or up to 1 M 1,2-PDO and 47.46 g/L biomass (Fig. 4C, D). In the LAD to *(S)*-lactate pathway model, all fucose was converted into biomass, which was consistent with the production of 1,2-PDO and biomass in the fermentation experiment (Fig. 4C). The EFMA results also showed that neither the LAD to *(S)*-lactate pathway nor the LAD to the pyruvaldehyde pathway could be maintained under anaerobic conditions because NAD+ could not be regenerated quickly enough (Fig. 4B). Instead, the yeast synthesized 1 M of ATP by converting 1 M of l-fucose into 1 M of 1,2-PDO and 1 M of pyruvate (Fig. 4B, D). This is consistent with the result that the yeast synthesized more 1,2-PDO than biomass when oxygen was limited (Fig. 3C, D).

*Saccharomyces boulardii* FCT, which can metabolize l-fucose, synthesizes small amounts of 1,2-PDO and large amounts of biomass through the *(S)*-lactaldehyde-*(S)*-lactate-pyruvate pathway under aerobic conditions; however, under anaerobic conditions, it synthesizes large amounts of 1,2-PDO to generate energy but does not produce much biomass. During fermentation under aerobic conditions, *S. boulardii* FCT used most of the l-fucose to synthesize biomass. This result was consistent with the predictions of multiple EFMA models for the LAD to *(S)*-lactate pathway when only l-fucose was available for biomass synthesis (Fig. 4C). In addition, other EFMA models for the *(S)*-lactate pathway used l-fucose for the simultaneous synthesis of relatively small amounts of biomass and large amounts of 1,2-PDO (Fig. 4C). On the other hand, the EFMA models for the pyruvaldehyde pathway predicted synthesis of at least 0.72 M of 1,2-PDO while metabolizing 1 M of l-fucose, which was markedly different from the fermentation data (Fig. 4C). These results indicate that *S. boulardii*, which metabolizes l-fucose under aerobic conditions, mostly synthesizes biomass along with a small amount of 1,2-PDO using the LAD to *(S)*-lactate pathway. Under microaerobic and strict anaerobic conditions, *S. boulardii* FCT metabolized l-fucose, and synthesized more 1,2-PDO, along with little or no biomass (Fig. 3C, D). The EFMA model showed that *S. boulardii* FCT metabolizes l-fucose under anaerobic conditions and produces 1,2-PDO, pyruvate, and ATP. When oxygen is limited, *S. boulardii* FCT may decrease biomass synthesis but increase 1,2-PDO synthesis by shifting the metabolic flux away from the LAD to *(S)*-lactate pathway and towards the production of 1,2-PDO and energy. Although pyruvate has not been quantified in anaerobic fermentation profiles, this could be because pyruvate is used in many different metabolic fluxes, as the molecule is a major metabolite in various metabolic processes, including glycolysis, gluconeogenesis, and pyruvic acid metabolism.

As *S. boulardii* FCT can consume l-fucose and synthesize energy and biological building blocks, it may have better metabolic activity than wild-type *S. boulardii* in the intestine. Although *S. boulardii* FCT showed a decrease in biomass synthesis under low-oxygen conditions, it still grew under microaerobic conditions (Fig. 3C). *S. boulardii* FCT also generated energy while synthesizing 1,2-PDO under strict anaerobic conditions (Figs. 3D and 4D). Because the oxygen concentration in the human intestine is very different depending on the location (apical mucosa closest to the lumen: 0.1–1%; intestinal wall: ~ 6%; colonic muscle wall: 7–10%) [40], *S. boulardii* FCT might be able to synthesize energy and biological building blocks from l-fucose under aerobic, microaerobic, and anaerobic conditions. Wild type *S. boulardii* can stay for 3 days in the intestine of a normal mouse, and can colonize the intestine of a gnotobiotic mouse [8]. The amount of l-fucose in the intestine is about 0.1 mmol, which can be continuously supplied by the host [31]. Assuming microaerobic conditions (Fig. 3C), 1 g DCW of *S. boulardii* FCT consumes 5.832 mmol of l-fucose, producing 0.144 g of biomass and 3.6 mmol of 1,2 PDO over 3 days. The growth rate of *S. boulardii* FCT on l-fucose may be insufficient to colonize the intestine before excretion. However, l-fucose still can be used for cell growth or survival by supplying energy and building blocks in the absence of other nutrient and as a supplementary energy source when *S. boulardii* FCT consumes other nutrients in the intestine. Animal studies would demonstrate the enhanced metabolic activity of *S. bouarldii* FCT in vivo. To do so, the integration of l-fucose metabolizing genes into the genome will be necessary to stably produce the heterologous enzymes in the intestine.

## Conclusion

*Saccharomyces boulardii* is a probiotic yeast that is widely used as a therapeutic agent for the treatment of intestinal diseases. In this study, we constructed *S. cerevisiae* and *S. boulardii* strains that metabolize l-fucose by selecting genes required for fucose metabolism based on GEM data and overexpressing fucose catabolic genes from *E. coli* and the transporter gene *HXT4* from *S. cerevisiae*. We also proposed mechanisms by which yeast strains metabolize l-fucose and generate biomass, 1,2-PDO, and energy under aerobic and anaerobic conditions. *S. boulardii* that can metabolize l-fucose in the intestine can continuously receive a carbon source from the host and could exert improved probiotic effects as a result. To our knowledge, this is the first study in which *S. cerevisiae* and *S. boulardii* strains capable of metabolizing l-fucose have been constructed. This strategy could also be used to enhance the metabolic activity of other probiotic microorganisms in the gut.

## Materials and methods

### Strains, plasmid construction, and culture conditions

The *fucU, fucA*, *fucK*, and *fucI* genes from *E. coli* K12 MG1655 and *HXT4* gene from *S. cerevisiae* CEN. PK2-1D were inserted into the plasmids pRS423GPD, pRS424GPD, and pRS426GPD [43]. *E. coli* DH5α was used for the construction and amplification of the plasmids. *S. cerevisiae* CEN. PK2-1D [44] and *S. boulardii* ATCC MYA-796 [36] were used. The plasmids were transformed into *S. cerevisiae* or *S. boulardii* using the lithium acetate/single-stranded carrier DNA/polyethylene glycol method [45]. *E. coli* strains were cultured in Luria–Bertani (LB) medium containing 100 µg/mL ampicillin at 37 °C and 200 rpm for amplification of plasmids. The *S. cerevisiae* or *S. boulardii* strains harboring the plasmids were cultured in yeast synthetic complete (YSC) medium containing 6.7 g/L yeast nitrogen base without amino acids, 20 g/L l-fucose (Biosynth Carbosynth, U.K.), and 0.70 g/L CSM-HIS-TRP-URA (MP Biomedicals). For tests under aerobic conditions, *S. cerevisiae* or *S. boulardii* were cultured in 100 mL fucose media in 250 mL flasks with shaking at 200 rpm. For tests under microaerobic conditions, *S. boulardii* was cultured in 100 mL fucose media in 160 mL serum bottles in a jar containing Anaero Pack-MicroAero (Mitsubishi Gas Chemical, Japan; O_2_ concentration of 6–12%, CO_2_ concentration of 5–8%) without shaking. After each sampling, the Anaero Pack was replaced. For tests under strict anaerobic conditions, *S. boulardii* was cultured in 100 mL fucose media in 160 mL serum bottles in an anaerobic chamber (Vinyl anaerobic chamber Type B; Coy Laboratory Products) without shaking. The atmosphere in the anaerobic chamber consisted of 90% nitrogen (v/v), 5% carbon dioxide (v/v), and 5% hydrogen (v/v).

### Analyses of extracellular metabolites

For the identification and quantification of extracellular metabolites, including d-glucose (Sigma-Aldrich), l-fucose (Sigma-Aldrich), and 1,2-PDO (Sigma-Aldrich), a high-performance liquid chromatography (HPLC) system equipped with a refractive index detector, Agilent 1100 (Agilent Technologies), and an Aminex HPX-87 H organic acid column (Bio-Rad) was used. We used a constant flow rate of 0.5 mL/min at 65 °C and 0.01 NH_2_SO_4_ as the mobile phase. The retention times of each metabolite were 17.373 min for d-glucose, 12.828 min for l-fucose, and 20.132 min for 1,2-PDO, respectively.

### Comparison of fucose transport efficiency among innate hexose transporters in *S. cerevisiae*

To investigate the fucose transport efficiency of each hexose transporter, the HXT-Null strain and the strains overexpressing HXT 1–7 were cultured in 5 mL of yeast extract and peptone (YP) media. The HXT-Null strain was cultured in YP containing ethanol (20 g/L) and the rest of the strains were cultured in YP containing glucose (20 g/L), and cells entering the intermediate exponential phase were harvested (OD 13/mL). The harvested cells were washed three times with distilled water (DW) and then cultured in 1 mL of YP with l-fucose (2 g/L) medium at 30 °C and 250 rpm for 4 h, and then 1 mL of cells was collected. The harvested cells were reconstituted in 500 µL of DW containing glass beads and vortexed for 1 h. The samples were centrifuged at 13,000 rpm for 30 min and 400 µL of the supernatant was collected and dried. The dried samples were reconstituted in 120 µL of DW, and the fucose concentration of each sample was analyzed by HPLC.

### GEM analysis to simulate the growth of ***S. cerevisiae*** that can metabolize l-fucose

The GEM for *S. cerevisiae* (yeast-GEM; version 8.5.0) [37] was used to simulate the growth of *S. cerevisiae* with the ability to metabolize l-fucose. To enable the simulation of fucose metabolism, β-l-fucose exchange, β-l-fucose transport, α-l-fucose exchange, α-l-fucose transport, fucose isomerase, fuculose kinase, fuculose-1-phosphate aldolase, 1,2-PDO oxidoreductase, and 1,2-PDO transport reactions were included in the model (Additional file 1). Extracellular β-l-fucose, cytoplasmic β-l-fucose, extracellular α-l-fucose, cytoplasmic α-l-fucose, cytoplasmic fuculose, cytoplasmic fuculose-1-phosphate, cytoplasmic 1,2-PDO, and extracellular 1,2-PDO were added to the model as the metabolites. MATLAB (version R2016b), Gurobi (version 8.0.1), and Cobra Toolbox (version 2.26) [46, 47] were used for GEM analysis. The MATLAB data file was uploaded to BioModels (MODEL2209060001).

### EFMA to elucidate the metabolic pathway of l-fucose under aerobic and anaerobic conditions

To elucidate the pathways of fucose metabolism in more detail, we modified the previously reported EFMA of *S. cerevisiae* [38]. Because this model contains many central carbon metabolic pathways, such as glycolysis, gluconeogenesis, the pentose phosphate pathway, and the TCA cycle, we introduced enzymes such as fucose isomerase, fuculose kinase, fuculose-1-phosphate aldolase, 1,2-PDO oxidoreductase, *(S)*-lactaldehyde dehydrogenase, *(S)*-lactic acid dehydrogenase, methylglyoxal reductase, and (D)-lactate dehydrogenase into the model. In addition, to obtain the absolute amount of the synthesized dried cell weight, we summed up the number moles of the building blocks (acetyl-CoA 0.269 M, α-ketoglutarate 0.11 M, erythrose-4-phosphate 0.003 M, 3-phosphoglyceric acid 0.006 M, glyceraldehyde-3-phosphate 0.001 M, phosphoenolpyruvate 0.006 M, pyruvate 0.018 M, ribulose-5-phosphate 0.003 M, glucose-6-phosphate 0.025 M, and oxaloacetate 0.01 M) required for cell growth and regarded it as the cell dry weight (15.74 g/L). The OD was converted to yeast dry cell weight based on a previously reported value [48].

## Supplementary Information

**Additional file 1.** Genome-scale metabolic model of Saccharomyces cerevisiae that metabolizes fucose. **Table S1.** Sequences of the plasmids constructed in this study.

## Data Availability

The datasets used and/or analysed during the current study are available from the corresponding author on reasonable request.
