# Supplementary material for: Strain engineering and metabolic flux analysis of a probiotic yeast Saccharomyces boulardii for metabolizing l-fucose, a mammalian mucin component
Source: Microb Cell Fact. 2022 Oct 7;21:204. doi: 10.1186/s12934-022-01926-x (PMC9541068; doi:10.1186/s12934-022-01926-x)
Supplement: Supplementary file 1 — Additional file 1. Genome-scale metabolic model of Saccharomyces cerevisiae that metabolizes fucose. Table S1. Sequences of the plasmids constructed in this study. [file 12934_2022_1926_MOESM1_ESM.docx]

**Supplementary Information**

**Table S1. Sequences of the plasmids constructed in this study**

**1. prs423GPD_*fucA (*pRS423GPD harboring *fucA* from *E. coli* K12 MG1655)**

gacgaaagggcctcgtgatacgcctatttttataggttaatgtcatgataataatggtttcttagtatgatccaatatcaaaggaaatgatagcattgaaggatgagactaatccaattgaggagtggcagcatatagaacagctaaagggtagtgctgaaggaagcatacgataccccgcatggaatgggataatatcacaggaggtactagactacctttcatcctacataaatagacgcatataagtacgcatttaagcataaacacgcactatgccgttcttctcatgtatatatatatacaggcaacacgcagatataggtgcgacgtgaacagtgagctgtatgtgcgcagctcgcgttgcattttcggaagcgctcgttttcggaaacgctttgaagttcctattccgaagttcctattctctagaaagtataggaacttcagagcgcttttgaaaaccaaaagcgctctgaagacgcactttcaaaaaaccaaaaacgcaccggactgtaacgagctactaaaatattgcgaataccgcttccacaaacattgctcaaaagtatctctttgctatatatctctgtgctatatccctatataacctacccatccacctttcgctccttgaacttgcatctaaactcgacctctacattttttatgtttatctctagtattactctttagacaaaaaaattgtagtaagaactattcatagagtgaatcgaaaacaatacgaaaatgtaaacatttcctatacgtagtatatagagacaaaatagaagaaaccgttcataattttctgaccaatgaagaatcatcaacgctatcactttctgttcacaaagtatgcgcaatccacatcggtatagaatataatcggggatgcctttatcttgaaaaaatgcacccgcagcttcgctagtaatcagtaaacgcgggaagtggagtcaggctttttttatggaagagaaaatagacaccaaagtagccttcttctaaccttaacggacctacagtgcaaaaagttatcaagagactgcattatagagcgcacaaaggagaaaaaaagtaatctaagatgctttgttagaaaaatagcgctctcgggatgcatttttgtagaacaaaaaagaagtatagattctttgttggtaaaatagcgctctcgcgttgcatttctgttctgtaaaaatgcagctcagattctttgtttgaaaaattagcgctctcgcgttgcatttttgttttacaaaaatgaagcacagattcttcgttggtaaaatagcgctttcgcgttgcatttctgttctgtaaaaatgcagctcagattctttgtttgaaaaattagcgctctcgcgttgcatttttgttctacaaaatgaagcacagatgcttcgttcaggtggcacttttcggggaaatgtgcgcggaacccctatttgtttatttttctaaatacattcaaatatgtatccgctcatgagacaataaccctgataaatgcttcaataatattgaaaaaggaagagtatgagtattcaacatttccgtgtcgcccttattcccttttttgcggcattttgccttcctgtttttgctcacccagaaacgctggtgaaagtaaaagatgctgaagatcagttgggtgcacgagtgggttacatcgaactggatctcaacagcggtaagatccttgagagttttcgccccgaagaacgttttccaatgatgagcacttttaaagttctgctatgtggcgcggtattatcccgtattgacgccgggcaagagcaactcggtcgccgcatacactattctcagaatgacttggttgagtactcaccagtcacagaaaagcatcttacggatggcatgacagtaagagaattatgcagtgctgccataaccatgagtgataacactgcggccaacttacttctgacaacgatcggaggaccgaaggagctaaccgcttttttgcacaacatgggggatcatgtaactcgccttgatcgttgggaaccggagctgaatgaagccataccaaacgacgagcgtgacaccacgatgcctgtagcaatggcaacaacgttgcgcaaactattaactggcgaactacttactctagcttcccggcaacaattaatagactggatggaggcggataaagttgcaggaccacttctgcgctcggcccttccggctggctggtttattgctgataaatctggagccggtgagcgtgggtctcgcggtatcattgcagcactggggccagatggtaagccctcccgtatcgtagttatctacacgacggggagtcaggcaactatggatgaacgaaatagacagatcgctgagataggtgcctcactgattaagcattggtaactgtcagaccaagtttactcatatatactttagattgatttaaaacttcatttttaatttaaaaggatctaggtgaagatcctttttgataatctcatgaccaaaatcccttaacgtgagttttcgttccactgagcgtcagaccccgtagaaaagatcaaaggatcttcttgagatcctttttttctgcgcgtaatctgctgcttgcaaacaaaaaaaccaccgctaccagcggtggtttgtttgccggatcaagagctaccaactctttttccgaaggtaactggcttcagcagagcgcagataccaaatactgtccttctagtgtagccgtagttaggccaccacttcaagaactctgtagcaccgcctacatacctcgctctgctaatcctgttaccagtggctgctgccagtggcgataagtcgtgtcttaccgggttggactcaagacgatagttaccggataaggcgcagcggtcgggctgaacggggggttcgtgcacacagcccagcttggagcgaacgacctacaccgaactgagatacctacagcgtgagctatgagaaagcgccacgcttcccgaagggagaaaggcggacaggtatccggtaagcggcagggtcggaacaggagagcgcacgagggagcttccagggggaaacgcctggtatctttatagtcctgtcgggtttcgccacctctgacttgagcgtcgatttttgtgatgctcgtcaggggggcggagcctatggaaaaacgccagcaacgcggcctttttacggttcctggccttttgctggccttttgctcacatgttctttcctgcgttatcccctgattctgtggataaccgtattaccgcctttgagtgagctgataccgctcgccgcagccgaacgaccgagcgcagcgagtcagtgagcgaggaagcggaagagcgcccaatacgcaaaccgcctctccccgcgcgttggccgattcattaatgcagctggcacgacaggtttcccgactggaaagcgggcagtgagcgcaacgcaattaatgtgagttacctcactcattaggcaccccaggctttacactttatgcttccggctcctatgttgtgtggaattgtgagcggataacaatttcacacaggaaacagctatgaccatgattacgccaagcgcgcaattaaccctcactaaagggaacaaaagctggagctctccaatttattctatccattagttgctgatatgtcccaccagccaacacttgatagtatctactcgccattcacttccagcagcgccagtagggttgttgagcttagtaaaaatgtgcgcaccacaagcctacatgactccacgtcacatgaaaccacaccgtggggccttgttgcgctaggaataggatatgcgacgaagacgcttctgcttagtaaccacaccacattttcagggggtcgatctgcttgcttcctttactgtcacgagcggcccataatcgcgctttttttttaaaaggcgcgagacagcaaacaggaagctcgggtttcaaccttcggagtggtcgcagatctggagactggatctttacaatacagtaaggcaagccaccatctgcttcttaggtgcatgcgacggtatccacgtgcagaacaacatagtctgaagaagggggggaggagcatgttcattctctgtagcagtaagagcttggtgataatgaccaaaactggagtctcgaaatcatataaatagacaatatattttcacacaatgagatttgtagtacagttctattctctctcttgcataaataagaaattcatcaagaacttggtttgatatttcaccaacacacacaaaaaacagtacttcactaaatttacacacaaaacaaatctagaactagtatgaaaaaaatcagcttaccgaaaattggtatccgcccggttattgacggtcgtcgcatgggtgttcgtgagtcgcttgaagaacaaacaatgaatatggcgaaagctacggccgcactgctgaccgagaaactgcgccatgcctgcggagctgccgtcgagtgtgtcatttccgatacctgtatcgcgggtatggctgaagccgctgcttgcgaagaaaaattcagcagtcagaatgtaggcctcaccattacggtaacgccttgctggtgctatggcagtgaaaccatcgacatggatccaacccgcccgaaggccatttggggctttaacggcactgaacgccccggcgctgtttacctggcagcggctctggcagctcacagccagaaaggcatcccagcattctccatttacggtcatgacgttcaggatgccgatgacacatcgattcctgccgatgttgaagaaaaactgctgcgctttgcccgcgccggtttggccgtcgccagcatgaaaggtaaaagctatctgtcgctgggcggcgtttcgatgggtatcgccggttccattgttgatcacaacttctttgaatcctggctgggaatgaaagtccaggcggtggatatgaccgaactgcgtcgccgtatcgatcagaagatttacgacgaagccgaattggaaatggcactggcctgggctgataaaaacttccgctatggcgaagatgaaaataacaaacagtatcaacgtaatgccgagcaaagccgcgcagttctgcgcgaaagtttactgatggcgatgtgtatccgcgacatgatgcaaggcaacagcaaactggccgatattggtcgcgtggaagaatcacttggctacaacgccatcgctgcgggcttccaggggcaacgtcactggaccgatcaatatcccaatggtgacaccgccgaagcgatcctcaacagttcatttgactggaatggcgtgcgcgaaccctttgtcgtggcgaccgaaaacgacagtcttaacggcgtggcaatgctaatgggtcaccagctcaccggcaccgctcaggtatttgccgatgtgcgtacctactggtcaccagaagcaattgagcgtgtaacggggcataaactggatggactggcagaacacggcatcatccatttgatcaactccggttctgctgcgctggacggttcctgtaaacaacgcgacagcgaaggtaacccgacgatgaagccacactgggaaatctctcagcaagaggctgacgcttgcctcgccgctaccgaatggtgcccggcgatccacgaatacttccgtggcggcggttactcttcccgcttccttaccgaaggcggcgtcccgttcaccatgactcgtgtcaacatcatcaaaggcctgggaccggtactgcaaatcgcggaaggctggagcgtggaattgccgaaggatgtgcatgacatcctcaacaaacgcaccaactcaacctggccaaccacctggtttgcaccgcgcctcaccggtaaagggccgtttacggatgtgtactcggtaatggcgaactggggcgctaaccatggggttctgaccatcggccacgttggcgcagactttatcactctcgcctccatgctgcgtatcccggtatgtatgcacaacgttgaagagaccaaagtgtatcgtccttctgcctgggctgcgcacggcatggatattgaaggccaggattaccgcgcttgccagaactacggtccgttgtacaagcgttaactcgagacaggccccttttcctttgtcgatatcatgtaattagttatgtcacgcttacattcacgccctcctcccacatccgctctaaccgaaaaggaaggagttagacaacctgaagtctaggtccctatttattttttttaatagttatgttagtattaagaacgttatttatatttcaaatttttcttttttttctgtacaaacgcgtgtacgcatgtaacattatactgaaaaccttgcttgagaaggttttgggacgctcgggtacccaattcgccctatagtgagtcgtattacgcgcgctcactggccgtcgttttacaacgtcgtgactgggaaaaccctggcgttacccaacttaatcgccttgcagcacatccccctttcgccagctggcgtaatagcgaagaggcccgcaccgatcgcccttcccaacagttgcgcagcctgaatggcgaatggcgcgacgcgccctgtagcggcgcattaagcgcggcgggtgtggtggttacgcgcagcgtgaccgctacacttgccagcgccctagcgcccgctcctttcgctttcttcccttcctttctcgccacgttcgccggctttccccgtcaagctctaaatcgggggctccctttagggttccgatttagtgctttacggcacctcgaccccaaaaaacttgattagggtgatggttcacgtagtgggccatcgccctgatagacggtttttcgccctttgacgttggagtccacgttctttaatagtggactcttgttccaaactggaacaacactcaaccctatctcggtctattcttttgatttataagggattttgccgatttcggcctattggttaaaaaatgagctgatttaacaaaaatttaacgcgaattttaacaaaatattaacgtttacaatttcctgatgcggtattttctccttacgcatctgtgcggtatttcacaccgcatagatccgtcgagttcaagagaaaaaaaaagaaaaagcaaaaagaaaaaaggaaagcgcgcctcgttcagaatgacacgtatagaatgatgcattaccttgtcatcttcagtatcatactgttcgtatacatacttactgacattcataggtatacatatatacacatgtatatatatcgtatgctgcagctttaaataatcggtgtcactacataagaacacctttggtggagggaacatcgttggtaccattgggcgaggtggcttctcttatggcaaccgcaagagccttgaacgcactctcactacggtgatgatcattcttgcctcgcagacaatcaacgtggagggtaattctgctagcctctgcaaagctttcaagaaaatgcgggatcatctcgcaagagagatctcctactttctccctttgcaaaccaagttcgacaactgcgtacggcctgttcgaaagatctaccaccgctctggaaagtgcctcatccaaaggcgcaaatcctgatccaaacctttttactccacgcgccagtagggcctctttaaaagcttgaccgagagcaatcccgcagtcttcagtggtgtgatggtcgtctatgtgtaagtcaccaatgcactcaacgattagcgaccagccggaatgcttggccagagcatgtatcatatggtccagaaaccctatacctgtgtggacgttaatcacttgcgattgtgtggcctgttctgctactgcttctgcctctttttctgggaagatcgagtgctctatcgctaggggaccaccctttaaagagatcgcaatctgaatcttggtttcatttgtaatacgctttactagggctttctgctctgtcatctttgccttcgtttatcttgcctgctcattttttagtatattcttcgaagaaatcacattactttatataatgtataattcattatgtgataatgccaatcgctaagaaaaaaaaagagtcatccgctaggggaaaaaaaaaaatgaaaatcattaccgaggcataaaaaaatatagagtgtactagaggaggccaagagtaatagaaaaagaaaattgcgggaaaggactgtgttatgacttccctgactaatgccgtgttcaaacgatacctggcagtgactcctagcgctcaccaagctcttaaaacgggaatttatggtgcactctcagtacaatctgctctgatgccgcatagttaagccagccccgacacccgccaacacccgctgacgcgccctgacgggcttgtctgctcccggcatccgcttacagacaagctgtgaccgtctccgggagctgcatgtgtcagaggttttcaccgtcatcaccgaaacgcgcga

**2. prs424GPD_*fucIfucU*: pRS424GPD harboring *fucI* and *fucU* from *E. coli* K12 MG1655**

gacgaaagggcctcgtgatacgcctatttttataggttaatgtcatgataataatggtttcttagtatgatccaatatcaaaggaaatgatagcattgaaggatgagactaatccaattgaggagtggcagcatatagaacagctaaagggtagtgctgaaggaagcatacgataccccgcatggaatgggataatatcacaggaggtactagactacctttcatcctacataaatagacgcatataagtacgcatttaagcataaacacgcactatgccgttcttctcatgtatatatatatacaggcaacacgcagatataggtgcgacgtgaacagtgagctgtatgtgcgcagctcgcgttgcattttcggaagcgctcgttttcggaaacgctttgaagttcctattccgaagttcctattctctagaaagtataggaacttcagagcgcttttgaaaaccaaaagcgctctgaagacgcactttcaaaaaaccaaaaacgcaccggactgtaacgagctactaaaatattgcgaataccgcttccacaaacattgctcaaaagtatctctttgctatatatctctgtgctatatccctatataacctacccatccacctttcgctccttgaacttgcatctaaactcgacctctacattttttatgtttatctctagtattactctttagacaaaaaaattgtagtaagaactattcatagagtgaatcgaaaacaatacgaaaatgtaaacatttcctatacgtagtatatagagacaaaatagaagaaaccgttcataattttctgaccaatgaagaatcatcaacgctatcactttctgttcacaaagtatgcgcaatccacatcggtatagaatataatcggggatgcctttatcttgaaaaaatgcacccgcagcttcgctagtaatcagtaaacgcgggaagtggagtcaggctttttttatggaagagaaaatagacaccaaagtagccttcttctaaccttaacggacctacagtgcaaaaagttatcaagagactgcattatagagcgcacaaaggagaaaaaaagtaatctaagatgctttgttagaaaaatagcgctctcgggatgcatttttgtagaacaaaaaagaagtatagattctttgttggtaaaatagcgctctcgcgttgcatttctgttctgtaaaaatgcagctcagattctttgtttgaaaaattagcgctctcgcgttgcatttttgttttacaaaaatgaagcacagattcttcgttggtaaaatagcgctttcgcgttgcatttctgttctgtaaaaatgcagctcagattctttgtttgaaaaattagcgctctcgcgttgcatttttgttctacaaaatgaagcacagatgcttcgttcaggtggcacttttcggggaaatgtgcgcggaacccctatttgtttatttttctaaatacattcaaatatgtatccgctcatgagacaataaccctgataaatgcttcaataatattgaaaaaggaagagtatgagtattcaacatttccgtgtcgcccttattcccttttttgcggcattttgccttcctgtttttgctcacccagaaacgctggtgaaagtaaaagatgctgaagatcagttgggtgcacgagtgggttacatcgaactggatctcaacagcggtaagatccttgagagttttcgccccgaagaacgttttccaatgatgagcacttttaaagttctgctatgtggcgcggtattatcccgtattgacgccgggcaagagcaactcggtcgccgcatacactattctcagaatgacttggttgagtactcaccagtcacagaaaagcatcttacggatggcatgacagtaagagaattatgcagtgctgccataaccatgagtgataacactgcggccaacttacttctgacaacgatcggaggaccgaaggagctaaccgcttttttgcacaacatgggggatcatgtaactcgccttgatcgttgggaaccggagctgaatgaagccataccaaacgacgagcgtgacaccacgatgcctgtagcaatggcaacaacgttgcgcaaactattaactggcgaactacttactctagcttcccggcaacaattaatagactggatggaggcggataaagttgcaggaccacttctgcgctcggcccttccggctggctggtttattgctgataaatctggagccggtgagcgtgggtctcgcggtatcattgcagcactggggccagatggtaagccctcccgtatcgtagttatctacacgacggggagtcaggcaactatggatgaacgaaatagacagatcgctgagataggtgcctcactgattaagcattggtaactgtcagaccaagtttactcatatatactttagattgatttaaaacttcatttttaatttaaaaggatctaggtgaagatcctttttgataatctcatgaccaaaatcccttaacgtgagttttcgttccactgagcgtcagaccccgtagaaaagatcaaaggatcttcttgagatcctttttttctgcgcgtaatctgctgcttgcaaacaaaaaaaccaccgctaccagcggtggtttgtttgccggatcaagagctaccaactctttttccgaaggtaactggcttcagcagagcgcagataccaaatactgtccttctagtgtagccgtagttaggccaccacttcaagaactctgtagcaccgcctacatacctcgctctgctaatcctgttaccagtggctgctgccagtggcgataagtcgtgtcttaccgggttggactcaagacgatagttaccggataaggcgcagcggtcgggctgaacggggggttcgtgcacacagcccagcttggagcgaacgacctacaccgaactgagatacctacagcgtgagctatgagaaagcgccacgcttcccgaagggagaaaggcggacaggtatccggtaagcggcagggtcggaacaggagagcgcacgagggagcttccagggggaaacgcctggtatctttatagtcctgtcgggtttcgccacctctgacttgagcgtcgatttttgtgatgctcgtcaggggggcggagcctatggaaaaacgccagcaacgcggcctttttacggttcctggccttttgctggccttttgctcacatgttctttcctgcgttatcccctgattctgtggataaccgtattaccgcctttgagtgagctgataccgctcgccgcagccgaacgaccgagcgcagcgagtcagtgagcgaggaagcggaagagcgcccaatacgcaaaccgcctctccccgcgcgttggccgattcattaatgcagctggcacgacaggtttcccgactggaaagcgggcagtgagcgcaacgcaattaatgtgagttacctcactcattaggcaccccaggctttacactttatgcttccggctcctatgttgtgtggaattgtgagcggataacaatttcacacaggaaacagctatgaccatgattacgccaagcgcgcaattaaccctcactaaagggaacaaaagctggagctctccaatttattctatccattagttgctgatatgtcccaccagccaacacttgatagtatctactcgccattcacttccagcagcgccagtagggttgttgagcttagtaaaaatgtgcgcaccacaagcctacatgactccacgtcacatgaaaccacaccgtggggccttgttgcgctaggaataggatatgcgacgaagacgcttctgcttagtaaccacaccacattttcagggggtcgatctgcttgcttcctttactgtcacgagcggcccataatcgcgctttttttttaaaaggcgcgagacagcaaacaggaagctcgggtttcaaccttcggagtggtcgcagatctggagactggatctttacaatacagtaaggcaagccaccatctgcttcttaggtgcatgcgacggtatccacgtgcagaacaacatagtctgaagaagggggggaggagcatgttcattctctgtagcagtaagagcttggtgataatgaccaaaactggagtctcgaaatcatataaatagacaatatattttcacacaatgagatttgtagtacagttctattctctctcttgcataaataagaaattcatcaagaacttggtttgatatttcaccaacacacacaaaaaacagtacttcactaaatttacacacaaaacaaatctagaactagtatggaacgaaataaacttgctcgtcagattattgacacttgcctggaaatgacccgcctgggactgaaccaggggacagcggggaacgtcagtgtacgttatcaggatgggatgctgattacgcctacaggcattccatatgaaaaactgacggagtcgcatattgtctttattgatggcaacggtaaacatgaggaaggaaagctcccctcaagcgaatggcgtttccatatggcagcctatcaaagcagaccggatgccaacgcggttgttcacaatcatgccgttcattgcacggcagtttccattcttaaccgatcgatccccgctattcactacatgattgcggcggctggcggtaattctattccttgcgcgccttatgcgacctttggaacacgcgaactttctgaacatgttgcgctggctctcaaaaatcgtaaggcaactttgttacaacatcatgggcttatcgcttgtgaggtgaatctggaaaaagcgttatggctggcgcatgaagttgaagtgctggcgcaactttacctgacgaccctggcgattacggacccggtgccagtgctgagcgatgaagagattgccgtagtgctggagaaattcaaaacctatgggttacgaattgaagagtaactcgagacaggccccttttcctttgtcgatatcatgtaattagttatgtcacgcttacattcacgccctcctcccacatccgctctaaccgaaaaggaaggagttagacaacctgaagtctaggtccctatttattttttttaatagttatgttagtattaagaacgttatttatatttcaaatttttcttttttttctgtacaaacgcgtgtacgcatgtaacattatactgaaaaccttgcttgagaaggttttgggacgctcgggtaccgagctctccaatttattctatccattagttgctgatatgtcccaccagccaacacttgatagtatctactcgccattcacttccagcagcgccagtagggttgttgagcttagtaaaaatgtgcgcaccacaagcctacatgactccacgtcacatgaaaccacaccgtggggccttgttgcgctaggaataggatatgcgacgaagacgcttctgcttagtaaccacaccacattttcagggggtcgatctgcttgcttcctttactgtcacgagcggcccataatcgcgctttttttttaaaaggcgcgagacagcaaacaggaagctcgggtttcaaccttcggagtggtcgcagatctggagactggatctttacaatacagtaaggcaagccaccatctgcttcttaggtgcatgcgacggtatccacgtgcagaacaacatagtctgaagaagggggggaggagcatgttcattctctgtagcagtaagagcttggtgataatgaccaaaactggagtctcgaaatcatataaatagacaatatattttcacacaatgagatttgtagtacagttctattctctctcttgcataaataagaaattcatcaagaacttggtttgatatttcaccaacacacacaaaaaacagtacttcactaaatttacacacaaaacaaatctagaactaatgctgaaaacaatttcgccgttaatttctcccgaactattgaaagtgctggcagagatgggacatggagatgaaattattttttccgatgctcactttcccgcccattcgatgggaccgcaggtgatccgcgctgatggcctgttggtgagcgacttgctccaggcgattatcccgttatttgaactggacagttatgcaccgccgctggtgatgatggcggcggtagaaggtgacactctcgatcctgaagtagaacgacgttaccgtaatgcgctttcactacaagccccgtgtcctgacatcatccgcatcaatcgttttgcgttttatgaacgggcgcaaaaagcctttgcgatcgttatcacaggcgaacgagcgaagtacgggaatattcttttaaaaaaaggggtaacaccgtaagtctcgagacaggccccttttcctttgtcgatatcatgtaattagttatgtcacgcttacattcacgccctcctcccacatccgctctaaccgaaaaggaaggagttagacaacctgaagtctaggtccctatttattttttttaatagttatgttagtattaagaacgttatttatatttcaaatttttcttttttttctgtacaaacgcgtgtacgcatgtaacattatactgaaaaccttgcttgagaaggttttgggacgctcgggtacccaattcgccctatagtgagtcgtattacgcgcgctcactggccgtcgttttacaacgtcgtgactgggaaaaccctggcgttacccaacttaatcgccttgcagcacatccccctttcgccagctggcgtaatagcgaagaggcccgcaccgatcgcccttcccaacagttgcgcagcctgaatggcgaatggcgcgacgcgccctgtagcggcgcattaagcgcggcgggtgtggtggttacgcgcagcgtgaccgctacacttgccagcgccctagcgcccgctcctttcgctttcttcccttcctttctcgccacgttcgccggctttccccgtcaagctctaaatcgggggctccctttagggttccgatttagtgctttacggcacctcgaccccaaaaaacttgattagggtgatggttcacgtagtgggccatcgccctgatagacggtttttcgccctttgacgttggagtccacgttctttaatagtggactcttgttccaaactggaacaacactcaaccctatctcggtctattcttttgatttataagggattttgccgatttcggcctattggttaaaaaatgagctgatttaacaaaaatttaacgcgaattttaacaaaatattaacgtttacaatttcctgatgcggtattttctccttacgcatctgtgcggtatttcacaccgcataggcaagtgcacaaacaatacttaaataaatactactcagtaataacctatttcttagcatttttgacgaaatttgctattttgttagagtcttttacaccatttgtctccacacctccgcttacatcaacaccaataacgccatttaatctaagcgcatcaccaacattttctggcgtcagtccaccagctaacataaaatgtaagctttcggggctctcttgccttccaacccagtcagaaatcgagttccaatccaaaagttcacctgtcccacctgcttctgaatcaaacaagggaataaacgaatgaggtttctgtgaagctgcactgagtagtatgttgcagtcttttggaaatacgagtcttttaataactggcaaaccgaggaactcttggtattcttgccacgactcatctccatgcagttggacgatatcaatgccgtaatcattgaccagagccaaaacatcctccttaggttgattacgaaacacgccaaccaagtatttcggagtgcctgaactatttttatatgcttttacaagacttgaaattttccttgcaataaccgggtcaattgttctctttctattgggcacacatataatacccagcaagtcagcatcggaatctagagcacattctgcggcctctgtgctctgcaagccgcaaactttcaccaatggaccagaactacctgtgaaattaataacagacatactccaagctgcctttgtgtgcttaatcacgtatactcacgtgctcaatagtcaccaatgccctccctcttggccctctccttttcttttttcgaccgaattaattcttaatcggcaaaaaaagaaaagctccggatcaagattgtacgtaaggtgacaagctatttttcaataaagaatatcttccactactgccatctggcgtcataactgcaaagtacacatatattacgatgctgtctattaaatgcttcctatattatatatatagtaatgtcgtttatggtgcactctcagtacaatctgctctgatgccgcatagttaagccagccccgacacccgccaacacccgctgacgcgccctgacgggcttgtctgctcccggcatccgcttacagacaagctgtgaccgtctccgggagctgcatgtgtcagaggttttcaccgtcatcaccgaaacgcgcga

**3. prs426GPD_*fucKHXT4:* pRS426GPD harboring *fucK* from *E. coli* K12 MG1655 and *HXT4* from *S. cerevisiae* CEN.PK2-1D**

gacgaaagggcctcgtgatacgcctatttttataggttaatgtcatgataataatggtttcttagtatgatccaatatcaaaggaaatgatagcattgaaggatgagactaatccaattgaggagtggcagcatatagaacagctaaagggtagtgctgaaggaagcatacgataccccgcatggaatgggataatatcacaggaggtactagactacctttcatcctacataaatagacgcatataagtacgcatttaagcataaacacgcactatgccgttcttctcatgtatatatatatacaggcaacacgcagatataggtgcgacgtgaacagtgagctgtatgtgcgcagctcgcgttgcattttcggaagcgctcgttttcggaaacgctttgaagttcctattccgaagttcctattctctagaaagtataggaacttcagagcgcttttgaaaaccaaaagcgctctgaagacgcactttcaaaaaaccaaaaacgcaccggactgtaacgagctactaaaatattgcgaataccgcttccacaaacattgctcaaaagtatctctttgctatatatctctgtgctatatccctatataacctacccatccacctttcgctccttgaacttgcatctaaactcgacctctacattttttatgtttatctctagtattactctttagacaaaaaaattgtagtaagaactattcatagagtgaatcgaaaacaatacgaaaatgtaaacatttcctatacgtagtatatagagacaaaatagaagaaaccgttcataattttctgaccaatgaagaatcatcaacgctatcactttctgttcacaaagtatgcgcaatccacatcggtatagaatataatcggggatgcctttatcttgaaaaaatgcacccgcagcttcgctagtaatcagtaaacgcgggaagtggagtcaggctttttttatggaagagaaaatagacaccaaagtagccttcttctaaccttaacggacctacagtgcaaaaagttatcaagagactgcattatagagcgcacaaaggagaaaaaaagtaatctaagatgctttgttagaaaaatagcgctctcgggatgcatttttgtagaacaaaaaagaagtatagattctttgttggtaaaatagcgctctcgcgttgcatttctgttctgtaaaaatgcagctcagattctttgtttgaaaaattagcgctctcgcgttgcatttttgttttacaaaaatgaagcacagattcttcgttggtaaaatagcgctttcgcgttgcatttctgttctgtaaaaatgcagctcagattctttgtttgaaaaattagcgctctcgcgttgcatttttgttctacaaaatgaagcacagatgcttcgttcaggtggcacttttcggggaaatgtgcgcggaacccctatttgtttatttttctaaatacattcaaatatgtatccgctcatgagacaataaccctgataaatgcttcaataatattgaaaaaggaagagtatgagtattcaacatttccgtgtcgcccttattcccttttttgcggcattttgccttcctgtttttgctcacccagaaacgctggtgaaagtaaaagatgctgaagatcagttgggtgcacgagtgggttacatcgaactggatctcaacagcggtaagatccttgagagttttcgccccgaagaacgttttccaatgatgagcacttttaaagttctgctatgtggcgcggtattatcccgtattgacgccgggcaagagcaactcggtcgccgcatacactattctcagaatgacttggttgagtactcaccagtcacagaaaagcatcttacggatggcatgacagtaagagaattatgcagtgctgccataaccatgagtgataacactgcggccaacttacttctgacaacgatcggaggaccgaaggagctaaccgcttttttgcacaacatgggggatcatgtaactcgccttgatcgttgggaaccggagctgaatgaagccataccaaacgacgagcgtgacaccacgatgcctgtagcaatggcaacaacgttgcgcaaactattaactggcgaactacttactctagcttcccggcaacaattaatagactggatggaggcggataaagttgcaggaccacttctgcgctcggcccttccggctggctggtttattgctgataaatctggagccggtgagcgtgggtctcgcggtatcattgcagcactggggccagatggtaagccctcccgtatcgtagttatctacacgacggggagtcaggcaactatggatgaacgaaatagacagatcgctgagataggtgcctcactgattaagcattggtaactgtcagaccaagtttactcatatatactttagattgatttaaaacttcatttttaatttaaaaggatctaggtgaagatcctttttgataatctcatgaccaaaatcccttaacgtgagttttcgttccactgagcgtcagaccccgtagaaaagatcaaaggatcttcttgagatcctttttttctgcgcgtaatctgctgcttgcaaacaaaaaaaccaccgctaccagcggtggtttgtttgccggatcaagagctaccaactctttttccgaaggtaactggcttcagcagagcgcagataccaaatactgtccttctagtgtagccgtagttaggccaccacttcaagaactctgtagcaccgcctacatacctcgctctgctaatcctgttaccagtggctgctgccagtggcgataagtcgtgtcttaccgggttggactcaagacgatagttaccggataaggcgcagcggtcgggctgaacggggggttcgtgcacacagcccagcttggagcgaacgacctacaccgaactgagatacctacagcgtgagctatgagaaagcgccacgcttcccgaagggagaaaggcggacaggtatccggtaagcggcagggtcggaacaggagagcgcacgagggagcttccagggggaaacgcctggtatctttatagtcctgtcgggtttcgccacctctgacttgagcgtcgatttttgtgatgctcgtcaggggggcggagcctatggaaaaacgccagcaacgcggcctttttacggttcctggccttttgctggccttttgctcacatgttctttcctgcgttatcccctgattctgtggataaccgtattaccgcctttgagtgagctgataccgctcgccgcagccgaacgaccgagcgcagcgagtcagtgagcgaggaagcggaagagcgcccaatacgcaaaccgcctctccccgcgcgttggccgattcattaatgcagctggcacgacaggtttcccgactggaaagcgggcagtgagcgcaacgcaattaatgtgagttacctcactcattaggcaccccaggctttacactttatgcttccggctcctatgttgtgtggaattgtgagcggataacaatttcacacaggaaacagctatgaccatgattacgccaagcgcgcaattaaccctcactaaagggaacaaaagctggagctctccaatttattctatccattagttgctgatatgtcccaccagccaacacttgatagtatctactcgccattcacttccagcagcgccagtagggttgttgagcttagtaaaaatgtgcgcaccacaagcctacatgactccacgtcacatgaaaccacaccgtggggccttgttgcgctaggaataggatatgcgacgaagacgcttctgcttagtaaccacaccacattttcagggggtcgatctgcttgcttcctttactgtcacgagcggcccataatcgcgctttttttttaaaaggcgcgagacagcaaacaggaagctcgggtttcaaccttcggagtggtcgcagatctggagactggatctttacaatacagtaaggcaagccaccatctgcttcttaggtgcatgcgacggtatccacgtgcagaacaacatagtctgaagaagggggggaggagcatgttcattctctgtagcagtaagagcttggtgataatgaccaaaactggagtctcgaaatcatataaatagacaatatattttcacacaatgagatttgtagtacagttctattctctctcttgcataaataagaaattcatcaagaacttggtttgatatttcaccaacacacacaaaaaacagtacttcactaaatttacacacaaaacaaatctagaactagtatgaaacaagaagttatcctggtactcgactgtggcgcgaccaatgtcagggccatcgcggttaatcggcagggcaaaattgttgcccgcgcctcaacgcctaatgccagcgatatcgcgatggaaaacaacacctggcaccagtggtctttagacgccattttgcaacgctttgctgattgctgtcggcaaatcaatagtgaactgactgaatgccacatccgcggtatcgccgtcaccacctttggtgtggatggcgctctggtagataagcaaggcaatctgctctatccgattattagctggaaatgtccgcgaacagcagcggttatggacaatattgaacggttaatctccgcacagcggttgcaggctatttctggcgtcggagcctttagtttcaatacgttatataagttggtgtggttgaaagaaaatcatccacaactgctggaacgcgcgcacgcctggctctttatttcgtcgctgattaaccaccgtttaaccggcgaattcactactgatatcacgatggccggaaccagccagatgctggatatccagcaacgcgatttcagtccgcaaattttacaagccaccggtattccacgccgactcttccctcgtctggtggaagcgggtgaacagattggtacgctacagaacagcgccgcagcaatgctcggcttacccgttggcataccggtgatttccgcaggtcacgatacccagttcgccctttttggcgctggtgctgaacaaaatgaacccgtgctctcttccggtacatgggaaattttaatggttcgcagcgcccaggttgatacttcgctgttaagtcagtacgccggttccacctgcgaactggatagccaggcagggttgtataacccaggtatgcaatggctggcatccggcgtgctggaatgggtgagaaaactgttctggacggctgaaacaccctggcaaatgttgattgaagaagctcgtctgatcgcgcctggcgcggatggcgtaaaaatgcagtgtgatttattgtcgtgtcagaacgctggctggcaaggagtgacgcttaataccacgcgggggcatttctatcgcgcggcgctggaagggttaactgcgcaattacagcgcaatctacagatgctggaaaaaatcgggcactttaaggcctctgaattattgttagtcggtggaggaagtcgcaacacattgtggaatcagattaaagccaatatgcttgatattccggtaaaagttctcgacgacgccgaaacgaccgtcgcaggagctgcgctgttcggttggtatggcgtaggggaatttaacagcccggaagaagcccgcgcacagattcattatcagtaccgttatttctacccgcaaactgaacctgaatttatagaggaagtgtgactcgagacaggccccttttcctttgtcgatatcatgtaattagttatgtcacgcttacattcacgccctcctcccacatccgctctaaccgaaaaggaaggagttagacaacctgaagtctaggtccctatttattttttttaatagttatgttagtattaagaacgttatttatatttcaaatttttcttttttttctgtacaaacgcgtgtacgcatgtaacattatactgaaaaccttgcttgagaaggttttgggacgctcgggtaccgagctctccaatttattctatccattagttgctgatatgtcccaccagccaacacttgatagtatctactcgccattcacttccagcagcgccagtagggttgttgagcttagtaaaaatgtgcgcaccacaagcctacatgactccacgtcacatgaaaccacaccgtggggccttgttgcgctaggaataggatatgcgacgaagacgcttctgcttagtaaccacaccacattttcagggggtcgatctgcttgcttcctttactgtcacgagcggcccataatcgcgctttttttttaaaaggcgcgagacagcaaacaggaagctcgggtttcaaccttcggagtggtcgcagatctggagactggatctttacaatacagtaaggcaagccaccatctgcttcttaggtgcatgcgacggtatccacgtgcagaacaacatagtctgaagaagggggggaggagcatgttcattctctgtagcagtaagagcttggtgataatgaccaaaactggagtctcgaaatcatataaatagacaatatattttcacacaatgagatttgtagtacagttctattctctctcttgcataaataagaaattcatcaagaacttggtttgatatttcaccaacacacacaaaaaacagtacttcactaaatttacacacaaaacaaatctagaactagtatgtctgaagaagctgcctatcaagaggatacagcagtccaaaatactccagctgatgctttgtcgccagttgaatccgattctaattccgctttgtctactccatccaacaaagctgaaagagatgacatgaaagatttcgacgagaatcacgaagaatctaataactacgttgaaattccaaagaagcccgcctccgcctacgttacagtttccatctgttgtttaatggttgctttcggtggttttgttttcggttgggatactggtaccatttctggttttgttgctcaaactgattttatcagaagatttggtatgaagcaccacgatggtacttattatttgtctaaggttagaactggtttaattgtctccattttcaacattggttgtgccattggtggtattattttagcaaaattaggtgatatgtatggtcgtaaaatgggtttgattgtcgttgttgtcatctacattatcggtatcattatccaaattgcctcaatcaacaagtggtaccaatatttcattggtagaattatctctggtttaggtgtcggtggtattgccgttttatctcctatgttgatttctgaagtctctccaaagcatattagaggtactttggtttcatgttaccaacttatgattactttgggtattttcttgggttactgtacaaactacggtaccaagacctacaccaattctgtccaatggagagttccattaggtctaggtttcgcttgggctttgtttatgattggtggtatgacattcgttccagaatctccacgttatttagttgaagtcggtaaaattgaagaagctaagcgttctattgctctttcaaataaggtcagcgcagacgatccagctgttatggctgaagtcgaagttgttcaagctacagttgaagctgaaaaattagctggtaatgcctcctggggtgaaatatttagcactaagactaaggttttccaacgtttgatcatgggtgctatgattcaatcattgcaacaattgacaggtgataactatttcttctattacggtactaccgttttcactgctgtcggtttggaagattcttttgaaacttctattgtcttgggtattgtcaactttgcttccacctttgttggtattttcttagtcgaaagatatggtcgtcgtagatgtttattatggggtgctgcttccatgacagcttgtatggttgttttcgcttctgttggtgttacaagattgtggccaaatggtaagaagaacgggtcttctaagggtgctggtaactgtatgattgtcttcacatgtttctacttattctgttttgccactacctgggctccaattccatttgttgttaactctgaaactttcccattgagagttaagtccaagtgtatggctattgctcaagcttgtaactggatctggggtttcttgattggtttctttactccatttatttcaggtgctattgatttctactacggttatgttttcatgggctgtttggtcttttcttacttctacgtcttcttcttcgttccagaaactaaaggtttgactttagaagaagttaacaccttatgggaagaaggtgttttgccatggaaatcaccttcttgggttccaccaaacaagagaggtactgactacaacgctgatgatctaatgcatgatgatcaaccattttacaagaagatgttcggaaaaaagtagctcgagacaggccccttttcctttgtcgatatcatgtaattagttatgtcacgcttacattcacgccctcctcccacatccgctctaaccgaaaaggaaggagttagacaacctgaagtctaggtccctatttattttttttaatagttatgttagtattaagaacgttatttatatttcaaatttttcttttttttctgtacaaacgcgtgtacgcatgtaacattatactgaaaaccttgcttgagaaggttttgggacgctcgggtacccaattcgccctatagtgagtcgtattacgcgcgctcactggccgtcgttttacaacgtcgtgactgggaaaaccctggcgttacccaacttaatcgccttgcagcacatccccctttcgccagctggcgtaatagcgaagaggcccgcaccgatcgcccttcccaacagttgcgcagcctgaatggcgaatggcgcgacgcgccctgtagcggcgcattaagcgcggcgggtgtggtggttacgcgcagcgtgaccgctacacttgccagcgccctagcgcccgctcctttcgctttcttcccttcctttctcgccacgttcgccggctttccccgtcaagctctaaatcgggggctccctttagggttccgatttagtgctttacggcacctcgaccccaaaaaacttgattagggtgatggttcacgtagtgggccatcgccctgatagacggtttttcgccctttgacgttggagtccacgttctttaatagtggactcttgttccaaactggaacaacactcaaccctatctcggtctattcttttgatttataagggattttgccgatttcggcctattggttaaaaaatgagctgatttaacaaaaatttaacgcgaattttaacaaaatattaacgtttacaatttcctgatgcggtattttctccttacgcatctgtgcggtatttcacaccgcatagggtaataactgatataattaaattgaagctctaatttgtgagtttagtatacatgcatttacttataatacagttttttagttttgctggccgcatcttctcaaatatgcttcccagcctgcttttctgtaacgttcaccctctaccttagcatcccttccctttgcaaatagtcctcttccaacaataataatgtcagatcctgtagagaccacatcatccacggttctatactgttgacccaatgcgtctcccttgtcatctaaacccacaccgggtgtcataatcaaccaatcgtaaccttcatctcttccacccatgtctctttgagcaataaagccgataacaaaatctttgtcgctcttcgcaatgtcaacagtacccttagtatattctccagtagatagggagcccttgcatgacaattctgctaacatcaaaaggcctctaggttcctttgttacttcttctgccgcctgcttcaaaccgctaacaatacctgggcccaccacaccgtgtgcattcgtaatgtctgcccattctgctattctgtatacacccgcagagtactgcaatttgactgtattaccaatgtcagcaaattttctgtcttcgaagagtaaaaaattgtacttggcggataatgcctttagcggcttaactgtgccctccatggaaaaatcagtcaagatatccacatgtgtttttagtaaacaaattttgggacctaatgcttcaactaactccagtaattccttggtggtacgaacatccaatgaagcacacaagtttgtttgcttttcgtgcatgatattaaatagcttggcagcaacaggactaggatgagtagcagcacgttccttatatgtagctttcgacatgatttatcttcgtttcctgcaggtttttgttctgtgcagttgggttaagaatactgggcaatttcatgtttcttcaacactacatatgcgtatatataccaatctaagtctgtgctccttccttcgttcttccttctgttcggagattaccgaatcaaaaaaatttcaaagaaaccgaaatcaaaaaaaagaataaaaaaaaaatgatgaattgaattgaaaagctgtggtatggtgcactctcagtacaatctgctctgatgccgcatagttaagccagccccgacacccgccaacacccgctgacgcgccctgacgggcttgtctgctcccggcatccgcttacagacaagctgtgaccgtctccgggagctgcatgtgtcagaggttttcaccgtcatcaccgaaacgcgcga

**4. prs426GPD_*fucK:* pRS426GPD harboring *fucK* from *E. coli* K12 MG1655**

gacgaaagggcctcgtgatacgcctatttttataggttaatgtcatgataataatggtttcttagtatgatccaatatcaaaggaaatgatagcattgaaggatgagactaatccaattgaggagtggcagcatatagaacagctaaagggtagtgctgaaggaagcatacgataccccgcatggaatgggataatatcacaggaggtactagactacctttcatcctacataaatagacgcatataagtacgcatttaagcataaacacgcactatgccgttcttctcatgtatatatatatacaggcaacacgcagatataggtgcgacgtgaacagtgagctgtatgtgcgcagctcgcgttgcattttcggaagcgctcgttttcggaaacgctttgaagttcctattccgaagttcctattctctagaaagtataggaacttcagagcgcttttgaaaaccaaaagcgctctgaagacgcactttcaaaaaaccaaaaacgcaccggactgtaacgagctactaaaatattgcgaataccgcttccacaaacattgctcaaaagtatctctttgctatatatctctgtgctatatccctatataacctacccatccacctttcgctccttgaacttgcatctaaactcgacctctacattttttatgtttatctctagtattactctttagacaaaaaaattgtagtaagaactattcatagagtgaatcgaaaacaatacgaaaatgtaaacatttcctatacgtagtatatagagacaaaatagaagaaaccgttcataattttctgaccaatgaagaatcatcaacgctatcactttctgttcacaaagtatgcgcaatccacatcggtatagaatataatcggggatgcctttatcttgaaaaaatgcacccgcagcttcgctagtaatcagtaaacgcgggaagtggagtcaggctttttttatggaagagaaaatagacaccaaagtagccttcttctaaccttaacggacctacagtgcaaaaagttatcaagagactgcattatagagcgcacaaaggagaaaaaaagtaatctaagatgctttgttagaaaaatagcgctctcgggatgcatttttgtagaacaaaaaagaagtatagattctttgttggtaaaatagcgctctcgcgttgcatttctgttctgtaaaaatgcagctcagattctttgtttgaaaaattagcgctctcgcgttgcatttttgttttacaaaaatgaagcacagattcttcgttggtaaaatagcgctttcgcgttgcatttctgttctgtaaaaatgcagctcagattctttgtttgaaaaattagcgctctcgcgttgcatttttgttctacaaaatgaagcacagatgcttcgttcaggtggcacttttcggggaaatgtgcgcggaacccctatttgtttatttttctaaatacattcaaatatgtatccgctcatgagacaataaccctgataaatgcttcaataatattgaaaaaggaagagtatgagtattcaacatttccgtgtcgcccttattcccttttttgcggcattttgccttcctgtttttgctcacccagaaacgctggtgaaagtaaaagatgctgaagatcagttgggtgcacgagtgggttacatcgaactggatctcaacagcggtaagatccttgagagttttcgccccgaagaacgttttccaatgatgagcacttttaaagttctgctatgtggcgcggtattatcccgtattgacgccgggcaagagcaactcggtcgccgcatacactattctcagaatgacttggttgagtactcaccagtcacagaaaagcatcttacggatggcatgacagtaagagaattatgcagtgctgccataaccatgagtgataacactgcggccaacttacttctgacaacgatcggaggaccgaaggagctaaccgcttttttgcacaacatgggggatcatgtaactcgccttgatcgttgggaaccggagctgaatgaagccataccaaacgacgagcgtgacaccacgatgcctgtagcaatggcaacaacgttgcgcaaactattaactggcgaactacttactctagcttcccggcaacaattaatagactggatggaggcggataaagttgcaggaccacttctgcgctcggcccttccggctggctggtttattgctgataaatctggagccggtgagcgtgggtctcgcggtatcattgcagcactggggccagatggtaagccctcccgtatcgtagttatctacacgacggggagtcaggcaactatggatgaacgaaatagacagatcgctgagataggtgcctcactgattaagcattggtaactgtcagaccaagtttactcatatatactttagattgatttaaaacttcatttttaatttaaaaggatctaggtgaagatcctttttgataatctcatgaccaaaatcccttaacgtgagttttcgttccactgagcgtcagaccccgtagaaaagatcaaaggatcttcttgagatcctttttttctgcgcgtaatctgctgcttgcaaacaaaaaaaccaccgctaccagcggtggtttgtttgccggatcaagagctaccaactctttttccgaaggtaactggcttcagcagagcgcagataccaaatactgtccttctagtgtagccgtagttaggccaccacttcaagaactctgtagcaccgcctacatacctcgctctgctaatcctgttaccagtggctgctgccagtggcgataagtcgtgtcttaccgggttggactcaagacgatagttaccggataaggcgcagcggtcgggctgaacggggggttcgtgcacacagcccagcttggagcgaacgacctacaccgaactgagatacctacagcgtgagctatgagaaagcgccacgcttcccgaagggagaaaggcggacaggtatccggtaagcggcagggtcggaacaggagagcgcacgagggagcttccagggggaaacgcctggtatctttatagtcctgtcgggtttcgccacctctgacttgagcgtcgatttttgtgatgctcgtcaggggggcggagcctatggaaaaacgccagcaacgcggcctttttacggttcctggccttttgctggccttttgctcacatgttctttcctgcgttatcccctgattctgtggataaccgtattaccgcctttgagtgagctgataccgctcgccgcagccgaacgaccgagcgcagcgagtcagtgagcgaggaagcggaagagcgcccaatacgcaaaccgcctctccccgcgcgttggccgattcattaatgcagctggcacgacaggtttcccgactggaaagcgggcagtgagcgcaacgcaattaatgtgagttacctcactcattaggcaccccaggctttacactttatgcttccggctcctatgttgtgtggaattgtgagcggataacaatttcacacaggaaacagctatgaccatgattacgccaagcgcgcaattaaccctcactaaagggaacaaaagctggagctctccaatttattctatccattagttgctgatatgtcccaccagccaacacttgatagtatctactcgccattcacttccagcagcgccagtagggttgttgagcttagtaaaaatgtgcgcaccacaagcctacatgactccacgtcacatgaaaccacaccgtggggccttgttgcgctaggaataggatatgcgacgaagacgcttctgcttagtaaccacaccacattttcagggggtcgatctgcttgcttcctttactgtcacgagcggcccataatcgcgctttttttttaaaaggcgcgagacagcaaacaggaagctcgggtttcaaccttcggagtggtcgcagatctggagactggatctttacaatacagtaaggcaagccaccatctgcttcttaggtgcatgcgacggtatccacgtgcagaacaacatagtctgaagaagggggggaggagcatgttcattctctgtagcagtaagagcttggtgataatgaccaaaactggagtctcgaaatcatataaatagacaatatattttcacacaatgagatttgtagtacagttctattctctctcttgcataaataagaaattcatcaagaacttggtttgatatttcaccaacacacacaaaaaacagtacttcactaaatttacacacaaaacaaatctagaactagtatgaaacaagaagttatcctggtactcgactgtggcgcgaccaatgtcagggccatcgcggttaatcggcagggcaaaattgttgcccgcgcctcaacgcctaatgccagcgatatcgcgatggaaaacaacacctggcaccagtggtctttagacgccattttgcaacgctttgctgattgctgtcggcaaatcaatagtgaactgactgaatgccacatccgcggtatcgccgtcaccacctttggtgtggatggcgctctggtagataagcaaggcaatctgctctatccgattattagctggaaatgtccgcgaacagcagcggttatggacaatattgaacggttaatctccgcacagcggttgcaggctatttctggcgtcggagcctttagtttcaatacgttatataagttggtgtggttgaaagaaaatcatccacaactgctggaacgcgcgcacgcctggctctttatttcgtcgctgattaaccaccgtttaaccggcgaattcactactgatatcacgatggccggaaccagccagatgctggatatccagcaacgcgatttcagtccgcaaattttacaagccaccggtattccacgccgactcttccctcgtctggtggaagcgggtgaacagattggtacgctacagaacagcgccgcagcaatgctcggcttacccgttggcataccggtgatttccgcaggtcacgatacccagttcgccctttttggcgctggtgctgaacaaaatgaacccgtgctctcttccggtacatgggaaattttaatggttcgcagcgcccaggttgatacttcgctgttaagtcagtacgccggttccacctgcgaactggatagccaggcagggttgtataacccaggtatgcaatggctggcatccggcgtgctggaatgggtgagaaaactgttctggacggctgaaacaccctggcaaatgttgattgaagaagctcgtctgatcgcgcctggcgcggatggcgtaaaaatgcagtgtgatttattgtcgtgtcagaacgctggctggcaaggagtgacgcttaataccacgcgggggcatttctatcgcgcggcgctggaagggttaactgcgcaattacagcgcaatctacagatgctggaaaaaatcgggcactttaaggcctctgaattattgttagtcggtggaggaagtcgcaacacattgtggaatcagattaaagccaatatgcttgatattccggtaaaagttctcgacgacgccgaaacgaccgtcgcaggagctgcgctgttcggttggtatggcgtaggggaatttaacagcccggaagaagcccgcgcacagattcattatcagtaccgttatttctacccgcaaactgaacctgaatttatagaggaagtgtgactcgagacaggccccttttcctttgtcgatatcatgtaattagttatgtcacgcttacattcacgccctcctcccacatccgctctaaccgaaaaggaaggagttagacaacctgaagtctaggtccctatttattttttttaatagttatgttagtattaagaacgttatttatatttcaaatttttcttttttttctgtacaaacgcgtgtacgcatgtaacattatactgaaaaccttgcttgagaaggttttgggacgctcgggtacccaattcgccctatagtgagtcgtattacgcgcgctcactggccgtcgttttacaacgtcgtgactgggaaaaccctggcgttacccaacttaatcgccttgcagcacatccccctttcgccagctggcgtaatagcgaagaggcccgcaccgatcgcccttcccaacagttgcgcagcctgaatggcgaatggcgcgacgcgccctgtagcggcgcattaagcgcggcgggtgtggtggttacgcgcagcgtgaccgctacacttgccagcgccctagcgcccgctcctttcgctttcttcccttcctttctcgccacgttcgccggctttccccgtcaagctctaaatcgggggctccctttagggttccgatttagtgctttacggcacctcgaccccaaaaaacttgattagggtgatggttcacgtagtgggccatcgccctgatagacggtttttcgccctttgacgttggagtccacgttctttaatagtggactcttgttccaaactggaacaacactcaaccctatctcggtctattcttttgatttataagggattttgccgatttcggcctattggttaaaaaatgagctgatttaacaaaaatttaacgcgaattttaacaaaatattaacgtttacaatttcctgatgcggtattttctccttacgcatctgtgcggtatttcacaccgcatagggtaataactgatataattaaattgaagctctaatttgtgagtttagtatacatgcatttacttataatacagttttttagttttgctggccgcatcttctcaaatatgcttcccagcctgcttttctgtaacgttcaccctctaccttagcatcccttccctttgcaaatagtcctcttccaacaataataatgtcagatcctgtagagaccacatcatccacggttctatactgttgacccaatgcgtctcccttgtcatctaaacccacaccgggtgtcataatcaaccaatcgtaaccttcatctcttccacccatgtctctttgagcaataaagccgataacaaaatctttgtcgctcttcgcaatgtcaacagtacccttagtatattctccagtagatagggagcccttgcatgacaattctgctaacatcaaaaggcctctaggttcctttgttacttcttctgccgcctgcttcaaaccgctaacaatacctgggcccaccacaccgtgtgcattcgtaatgtctgcccattctgctattctgtatacacccgcagagtactgcaatttgactgtattaccaatgtcagcaaattttctgtcttcgaagagtaaaaaattgtacttggcggataatgcctttagcggcttaactgtgccctccatggaaaaatcagtcaagatatccacatgtgtttttagtaaacaaattttgggacctaatgcttcaactaactccagtaattccttggtggtacgaacatccaatgaagcacacaagtttgtttgcttttcgtgcatgatattaaatagcttggcagcaacaggactaggatgagtagcagcacgttccttatatgtagctttcgacatgatttatcttcgtttcctgcaggtttttgttctgtgcagttgggttaagaatactgggcaatttcatgtttcttcaacactacatatgcgtatatataccaatctaagtctgtgctccttccttcgttcttccttctgttcggagattaccgaatcaaaaaaatttcaaagaaaccgaaatcaaaaaaaagaataaaaaaaaaatgatgaattgaattgaaaagctgtggtatggtgcactctcagtacaatctgctctgatgccgcatagttaagccagccccgacacccgccaacacccgctgacgcgccctgacgggcttgtctgctcccggcatccgcttacagacaagctgtgaccgtctccgggagctgcatgtgtcagaggttttcaccgtcatcaccgaaacgcgcga
